# *Haloterrigena* sp. Strain SGH1, a Bacterioruberin-Rich, Perchlorate-Tolerant Halophilic Archaeon Isolated From Halite Microbial Communities, Atacama Desert, Chile

**DOI:** 10.3389/fmicb.2020.00324

**Published:** 2020-03-05

**Authors:** Nataly Flores, Sebastián Hoyos, Mauricio Venegas, Alexandra Galetović, Lidia M. Zúñiga, Francisca Fábrega, Bernardo Paredes, Camila Salazar-Ardiles, Claudia Vilo, Carmen Ascaso, Jacek Wierzchos, Virginia Souza-Egipsy, Jorge E. Araya, Ramón Alberto Batista-García, Benito Gómez-Silva

**Affiliations:** ^1^Laboratory of Biochemistry, Biomedical Department and Centre for Biotechnology and Bioengineering, Universidad de Antofagasta, Antofagasta, Chile; ^2^Department Biogeochemistry and Microbial Ecology, National Museum of Natural Sciences – Spanish National Research Council, Madrid, Spain; ^3^Department of Macromolecular Physics, Institute of Material Structure – Spanish National Research Council, Madrid, Spain; ^4^Laboratory of Molecular Parasitology, Department of Medical Technology and Centre for Biotechnology and Bioengineering, Universidad de Antofagasta, Antofagasta, Chile; ^5^Centro de Investigación en Dinámica Celular, Instituto de Investigación en Ciencias Básicas y Aplicadas, Universidad Autónoma del Estado de Morelos, Cuernavaca, Mexico

**Keywords:** antioxidant, archaea, Atacama, bacterioruberin, Haloterrigena, halites, perchlorate, toxicity

## Abstract

An extreme halophilic archaeon, strain SGH1, is a novel microorganism isolated from endolithic microbial communities colonizing halites at Salar Grande, Atacama Desert, in northern Chile. Our study provides structural, biochemical, genomic, and physiological information on this new isolate living at the edge of the physical and chemical extremes at the Atacama Desert. SGH1 is a Gram-negative, red-pigmented, non-motile unicellular coccoid organism. Under the transmission electron microscope, strain SGH1 showed an abundant electro-dense material surrounding electron-lucent globular structures resembling gas vacuoles. Strain SGH1 showed a 16S rRNA gene sequence with a close phylogenetic relationship to the extreme halophilic archaea *Haloterrigena turkmenica* and *Haloterrigena salina* and has been denominated *Haloterrigena* sp. strain SGH1. Strain SGH1 grew at 20–40°C (optimum 37°C), at salinities between 15 and 30% (w/v) NaCl (optimum 25%) and growth was improved by addition of 50 mM KCl and 0.5% w/v casamino acids. Growth was severely restricted at salinities below 15% NaCl and cell lysis is avoided at a minimal 10% NaCl. Maximal concentrations of magnesium chloride and sodium or magnesium perchlorates that supported SGH1 growth were 0.5 and 0.15M, respectively. *Haloterrigena* sp. strain SGH1 accumulates bacterioruberin (BR), a C_50_ xanthophyll, as the major carotenoid. Total carotenoids in strain SGH1 amounted to nearly 400 μg BR per gram of dry biomass. Nearly 80% of total carotenoids accumulated as geometric isomers of BR: all-*trans*-BR (50%), 5-*cis*-BR (15%), 9-*cis*-BR (10%), 13-*cis*-BR (4%); other carotenoids were dehydrated derivatives of BR. Carotenogenesis in SGH1 was a reversible and salt-dependent process; transferring BR-rich cells grown in 25% (w/v) NaCl to 15% (w/v) NaCl medium resulted in depigmentation, and BR content was recovered after transference and growth of unpigmented cells to high salinity medium. Methanol extracts and purified BR isomers showed an 8–9-fold higher antioxidant activity than Trolox or β-carotene. Both, plasma membrane integrity and mitochondrial membrane potential measurements under acute 18-h assays showed that purified BR isomers were non-toxic to cultured human THP-1 cells.

## Introduction

The Atacama Desert is considered to be the driest and oldest dryland on Earth (McKay et al., 2003; Navarro-González et al., 2003). The microbiological evidence accumulated during the last two decades has provided the base for a new paradigmatic view of Atacama, from a sterile territory to a region, today, with a rich microbiota adapted to the prevalent extreme physical and chemical conditions of this hyperarid desert (Bull et al., 2016; Cordero et al., 2016; Gómez-Silva, 2018; Wierzchos et al., 2018). Due to severe liquid water limitations, desiccation in Atacama is the key environmental factor that seriously restricts abundance and diversity of microbial life. The high solar radiation in Atacama accompanies desiccation in a synergic fashion (Wierzchos et al., 2015; Cockell et al., 2017; Gómez-Silva, 2018; Meslier et al., 2018). However, Atacama contains a rich genetic resource of extremophiles and extremotolerant microorganisms (Bull et al., 2016; Gómez-Silva et al., 2019).

Lithobiontic colonization can be considered as one of the most successful strategies for microbial survival under the extreme desiccation and high solar radiation on the Atacama Desert. Lithic habitats provide one of the most efficient niches to sustain microbial life by acting as a filter to solar and ultraviolet radiations, as a liquid water harvesting device from fogs and dew events, by salt deliquescence and capillary condensation (Davila et al., 2008; Wierzchos et al., 2015, 2018; Cockell et al., 2017; Gómez-Silva, 2018; Meslier et al., 2018; Uritskiy et al., 2019). In Atacama, lithic habitats such as halite nodules are colonized by endolithic and epilithic microbial consortia that include members of the three domains of life and viruses (de los Ríos et al., 2010; Gómez-Silva, 2010, 2018; Robinson et al., 2015; Crits-Christoph et al., 2016a, b; Wierzchos et al., 2018).

Salar Grande is a coastal endorheic basin located at the eastern slopes of the Coastal Range in northern Chile and subjected to high solar insolation and desiccation for millions of years (Sánchez-García et al., 2018). This fossil saltflat is a rich sodium chloride deposit where lithobiontic communities live inside and at the surface of halite nodules, with a surprising and still non-culturable microbial diversity (Robinson et al., 2015; Crits-Christoph et al., 2016a, b; Gómez-Silva, 2018). Our group has been able to grow the first microorganism isolated from these endolithic consortia colonizing halites at Salar Grande. This isolate has been denominated *Haloterrigena* sp. strain SGH1 and it is an extreme halophilic archaeon with a high content of the carotenoid bacterioruberin (Hoyos, 2016; Flores, 2017). In this work, we provide structural and biochemical information on strain SGH1 and on the identification, content, antioxidant properties and toxicity of its BR isomers.

## Materials and Methods

### Sampling Site

Salar Grande, with an approximate surface of 500 km^2^, is located at the eastern slopes of the Coastal Range of the Tarapacá Region in northern Chile, at 650 m of altitude and 80 km south of Iquique City. Halite nodules were collected at the north-east border of Salar Grande (20°56′ S; 70°00′ W). Collection and further work were carried out aseptically with all materials previously sterilized.

### Isolation, Growth, and Maintenance of Strain SGH1

Halites samples were broken open to expose the endolithic colonization which can be observed as a greenish line that runs parallel at few mm below the rock surface (Gómez-Silva, 2018). Using a sterilized metal tool, a careful scrapping of the colonized zones was conducted to avoid contamination by fragments from the rock surface. A 300-mg sample was added to liquid Z8 growth medium (NIVA, 1976), modified to contain 5 mM NaNO_3_, 50 mM KCl, with NaCl at concentrations between 0 and 30% w/v (medium Z8-NK). Compatible organic solutes (L-proline, trehalose, or betaine) were also added to the Z8-NK medium at 10 mM final concentration. Cultures were incubated at 30°C, in a rotary incubator (Zichen ZWWY-100B) at 120 rpm, under constant illumination with white fluorescent light (34 μmoles m^–2^ s^–1^). After 2–3 weeks, growth was observed in cultures growing in Z8-NK containing 25% NaCl and 10 mM L-proline, as an evident increase in turbidity. Aliquots were transferred to 3% agar plates prepared in Z8-NK plus 25% NaCl and 10 mM L-proline and incubated until colonies were observed. Light microscopy observations, before or after Gram staining, confirmed the presence of only one type of microorganism in the isolated colonies. The isolated microorganism was denominated strain SGH1. SGH1 growth improvement was obtained after L-proline was replaced by 0.5% w/v casamino acids. This new medium, Z8-MOD at 25% NaCl, was used for maintenance and biomass production. Strain SGH1 was conserved using glycerol 25% at −80°C.

### Transmission Electron Microscopy (TEM) of SGH1

Aliquots of liquid cultures and agar colonies were fixed 3 h at 5°C with 3% glutaraldehyde (EMS grade) in 0.1 mM cacodylate buffer. Samples were washed several times by centrifugation and pelleted in fresh sterile agar. Pellets were post-fixed with 1% (w/v) osmium tetroxide for 5 h at room temperature. Dehydration included a step with 70% ethanol with 2% uranyl acetate to increase contrast of intracellular components. Samples were infiltrate with LR-White resin and polymerized using gelatin capsules at 60°C for 24 h. An Ultracut E ultramicrotome (Reichert-Jung) was used for preparation of ultrathin 80 nm sections that were placed on copper grids and contrasted with lead citrate. Grids were examined at 80 kV with a Leo 910 (Oberkochen, Germany) transmission electron microscope equipped with a Gatan BioScan 792 camera (Warrendale, United States).

### Effect of Salinity, pH, Temperature, Magnesium, and Perchlorate on SGH1 Growth

Strain SGH1 (aliquots of 0.5 mL; A^660^: 0.1) was cultured in 25 mL of liquid medium Z8-MOD at different final salinities from 0 to 30% w/v NaCl. Once the optimal salinity was determined, the SGH1 cultures were grown at various pH (from 6 to 10) and temperatures (from 20 to 50°C) in order to define optimal growth conditions. SGH1 growth was followed as increments in turbidity at 660 nm and doubling time were computed at the exponential phase of growth. Tolerance of strain SGH1 to MgCl_2_ x 6H_2_O (50 to 500 mM), to sodium or magnesium perchlorate (50 to 300 mM) was conducted in independent growth experiments, in triplicates, in medium Z8-MOD. The specific growth rate for each condition was computed after 96 h of growth.

### Carotenoid Extraction

Strain SGH1 grown in Z8-MOD medium was harvested at the early stationary phase of growth by centrifugation (15 min at 5,000 rpm, rotor Sorvall SS-34). Cells were washed with fresh growth medium at a final concentration of 10% (w/v) NaCl and, cells were recovered by centrifugation (4,000 rpm for 10 min, IEC centrifuge Centra CL2). The cell pellet was lyophilized and the dried biomass was exhaustively extracted with 100% methanol (HPLC grade) at 4°C, under dim light, in order to obtain a fraction enriched in total carotenoids. The methanolic extracts were clarified by centrifugation (4,000 rpm for 10 min, IEC centrifuge Centra CL2) and the uncolored cell residues were discarded. The methanolic extract was dried by N_2_ bubbling or using a rotavapor, the pigment residue was suspended 100% methanol, filtered with in a PTFE filter (0.22 um) and finally stored in amber bottles at 4°C.

### Fractionation, Quantitation, and Identification of Carotenoids

Absorption spectrum (350–600 nm) of carotenoid extracts were obtained in a Shimadzu spectrophotometer UV-1601 and absorbance values at 494 nm were used to compute total SGH1 carotenoids concentration in SGH1 methanol extracts, using the following equation and the extinction coefficient for BR in methanol (2,660 g%^–1^ cm^–1^) (Mandelli et al., 2012; De la Vega et al., 2016):

$$C\,\left(u\,g/m\,l\right)=\frac{{\text{A}}^{494}\times 10^{6}}{\varepsilon_{1\,c\,m}^{1\%}\times 100}$$

ethanol extracts (50 μL) were injected to a Shimatzu Hitachi CL-20A HPLC system containing a C18 reverse phase column (Merck 1.50477.0001, LiChrospher 100 RP-18.5 μm) as stationary phase. For elution, the mobile phase used was methanol/acetonitrile/water (85:10:5, v/v) at a flow rate of 1.0 mL/min during 12 min. The collection of fractions was conducted after programming the HPLC system software to start and finish at specific retention times to avoid contamination among the collected peaks. The more abundant HPLC fractions were recovered, dried under N_2_ and dissolved in 100% methanol. Each recovered HPLC fraction (10 μL) was injected in a Thermo Scientific Dionex Ultimate-3000 UHPLC system with a C18 column (Ultra AQ-C18, 3 μm, 100 mm × 2.1 mm); the mobile phase for elution was a methanol/acetonitrile/water gradient (20:60:20, v/v) for 2 min, 30:70:0 (v/v) for 10 min, 50:50:0 (v/v) for 15 min, and 90:10:0 (v/v) for 20 min, at 25°C, at a flow rate of 1.0 mL per min. Data analysis was managed with the Xcalibur 2.3 software (Thermo Fisher Scientific). Absorbance spectra were conducted at 494 nm. The UHPLC chromatographer was coupled to a high-resolution mass spectrometer Q Exactive (Thermo Fisher Scientific) controlled by the Q Exactive 2.0 SP 2 software (Thermo Fisher Scientific). Carotenoid ionization was conducted with an Electrospray Ionization Source at 400°C and 2,500 V, under nitrogen (Genus NM32LA Peak Scientific) to generate ionized fragments. The scanning of positively ionized fragments was conducted at 100 to 1,000 m/z. Caffeine, *N*-butylamine, buspirone, sodium dodecyl sulfate, and taurocholate served as standards (Sigma-Aldrich). The scanning of positively ionized fragments was conducted at 100 to 1,000 m/z.

C-50 carotenoid identification from strain *Haloterrigena* sp. SGH1 was conducted by data analyses of Raman spectroscopy, chromatographic migrations, UV-Vis spectra, spectral fine structure and MS fragmentation patterns (Fong et al., 2001; Mandelli et al., 2012; Jehlička et al., 2014; De la Vega et al., 2016; Dina et al., 2017).

### Raman Spectroscopy

Freeze-dried SGH1 cells were suspended on glass slides or nylon filters (0.2 μm) using a 25% w/v NaCl solution. Next, the samples were dried at room temperature for 24 h in darkness. Micro-Raman analyses of upper region of the dried samples were performed on a multichannel Raman DXL Thermo Fischer spectrometer coupled with a Peltier-cooled (−50°C) CCD detector and equipped with 10x objective. The grating with 900 lines/mm was used. Excitation was provided by the 523 nm Argon laser. Spectrograph slit aperture was 25 μm. To achieve enhanced signal-to-noise ratios, 44 scans were accumulated, each of 2.70 s exposure time with a laser power of 8.0 mW at the samples. The background exposures were 512. Spectra were recorded with a spectral resolution of 1.9 cm^–1^ over the wavenumber range 150 and 2.000 cm^–1^. Seven to 10 measurements were obtained on the investigated pellet. Calibration of the apparatus was made before measurements using a calibration tool for the Raman DXL Thermo Fischer spectrometer. Raman spectra were not subjected to data manipulation or processing techniques and were reported as collected. OMNIC 8.3 software was used for data visualization.

### Antioxidative Activity of SGH1 Carotenoids

Three assays were employed to evaluate antioxidative activity of the methanolic extracts and fractions purified by HPLC chromatography.

#### Fenton Reaction on Plasmid DNA

This assay involves the formation of hydroxyl radicals from H_2_O_2_ in the presence of ferrous ions and, oxidative damage to DNA can be observed by a differential electrophoretic mobility of relaxed and supercoiled molecules (Ishikawa et al., 2002). The assay was modified to a final volume of 50 μL and included 10 μL of a solution containing 423 ng/μL of pUC19 plasmid DNA. Plasmid pUC19 was incubated with Fenton’s reagent in the presence or absence of two doses (106 and 212 μg/mL) of carotenoids from the SGH1 methanolic extracts. Autoclaved deionized water was included in assays as negative control. The positive control included the Fenton reagent (15 μL of 3% H_2_O_2_ plus 15 μL 100 μM FeCl_2_). Oxidized DNA decreases its electrophoretic mobility when compared with intact non-oxidized supercoiled DNA. Protection of plasmid DNA to oxidation was evaluated with 5 μL of Trolox (50 μg/μL), 5 μL of β-carotene (50 μg/μL) or 5 μL of SGH1 extract (final concentrations of 0.001 or 0.002 μg/μL). The assays were incubated at 37°C during 30 min and stopped by adding 1.0 μL of 5 mM EDTA. Electrophoresis of intact or damaged plasmid DNA was carried out at room temperature on 0.8% agarose gels for 40 min at 80 mV. The gels were stained with ethidium bromide, dried and bands densitometry was estimated by ImageJ, an open source image processing program.^1^

#### ABTS Assay (2,2′-Azinobis-(3-Ethyl-Benzothiazole-6-Sulfonic Acid))

A solution of 7 mM ABTS dissolved in water was mixed with 2.45 mM potassium persulfate in a 1:1 (v/v) ratio during 16 h to generate the radical ABTS^*+^. This mix was diluted with 70% ethanol until the solution reached an absorbance of 0.7 ± 0.02 at 734 nm. The assay included 1.98 mL of the ABTS^*+^ solution plus 0.02 mL of a carotenoid solution and it was incubated during 7 min at room temperature, in darkness. A decrease in absorbance at 734 nm was used to compute the antioxidant activity of the carotenoid samples, expressed as percentage of inhibition of the oxidation of ABTS^*+^. The antioxidant activity of SGH1 carotenoids was finally expressed as TEAC (Trolox equivalent antioxidant capacity) and IC_50_ (concentration of carotenoids expressed as μg/ml, needed to scavenge 50% of ABTS^*+^) (Re et al., 1999; Mandelli et al., 2012; Konan et al., 2016). Each independent experiment evaluated 4–5 different concentrations of antioxidant in assays in triplicate.

#### FRAP Assay (Ferric Ion Reducing Antioxidant Power)

The reducing power of SGH1 carotenoids was done mixing SGH1 carotenoid samples (100 μL) with a freshly prepared solution containing TPTZ-Fe^+3^ (ferric 2,4,6-tripyridyl-s-triazine) which was prepared with 10 mM TPTZ, 20 mM FeCl_3_ x 6 H_2_O and 0.3M acetate buffer, pH 3.6, at a ratio 1:1:10 (v/v). The assay was conducted during 30 min at room temperature, in darkness. Absorbance at 593 nm was computed to determine the antioxidant activity of carotenoids with respect to Trolox (Albiochem) (Benzie and Strain, 1996; Pulido et al., 2000). The antioxidant activity of SGH1 carotenoids was finally expressed as TEAC values. Each independent experiment evaluated 4–5 different concentrations of antioxidant in assays in triplicate.

### Cellular Toxicity of SGH1 Carotenoids

THP-1 cell line (ATCC^®^ 30-2001) derived from the blood of a 1-year-old boy with acute monocytic leukemia (Tsuchiya et al., 1980) was used to analyze cellular toxicity of methanolic extracts and carotenoids from *Haloterrigena* sp. strain SGH1. THP-1 cells were grown in RPMI-1640 medium (Gibco Cat. 11875-085) containing 10% fetal bovine serum (Gibco Cat. 11875085) at 37°C, in an atmosphere of 5% CO_2_ in air. HP-1 cells were selected for these studies since cytotoxicity and other cellular responses can be easily and rapidly conducted by flow cytometry; also, they are non-adherent easily cultured cells and, as bloodstream circulating cells, they are a reasonable experimental model for exposure to high concentrations of novel compounds that could eventually be used in biomedical applications (Pick et al., 2004).

#### Plasma Membrane Integrity Assay

Cell viability was evaluated by dye exclusion using a membrane impermeant compound. Propidium iodide (PI; Molecular Probes Cat. P1304MP) is a membrane impermeant fluorogenic dye generally excluded from viable cells (Darzynkiewicz et al., 1992). PI binds to DNA and provides fluorescence to death cells and can be quantified by flow cytometry (Riccardi and Nicoletti, 2006). Cellular toxicity of methanol extracts, fractions FI, FII, FIII, FIV, and FV from strain SGH1 against THP-1 cells was evaluated using PI and measured by flow cytometry (BD FACSJazz cytometer). Antimycin A (SIGMA Cat. A8674), an inhibitor of mitochondrial electron transport (complex III), was used as a positive control. THP-1 cells (5 × 10^5^ cells/mL) were starved for 24 h in FluoroBrite DMEM medium (Gibco Cat. A18967-01), free of fetal bovine serum and glutathione. Next, cells were incubated with 0.5% (v/v) methanol (MeOH; control), methanol extract (500 μg/mL), purified Fractions I, II, III, IV, or V (500 μg/mL), 0.5% (v/v) ethanol (EtOH; Antimycin control) or Antimycin A (30 μM), as final concentrations in the assays. Eighteen hours after treatment, cells were incubated with 2.4 μM PI during 15 min. PI positive cells were measured by flow cytometry using an excitation laser beam at 488 and 585 nm detector. Results were expressed as percentage of viable cells. The assays were done in four independent experiments.

### Mitochondrial Activity Assay

Cell viability was also evaluated by measurements of the mitochondrial membrane potential (ΔΨm), which can be lost in cells undergoing apoptosis or necrosis (Bradham et al., 1998; Lemasters et al., 1998; Zorova et al., 2018). MitoStatus Red (MTRed; BD Pharmigen^TM^ Cat. 564697) is a cationic, lipophilic and fluorescent dye that is readily sequestered by active mitochondria of healthy cells, but not within mitochondria that have lost its ΔΨm (Rottenberg and Wu, 1998). Cellular toxicity of methanol extracts, fractions FI, FII, FIII, FIV, and FV from strain SGH1 against THP-1 cells was evaluated using MTRed and measured by flow cytometry. THP-1 cells (5 × 10^5^ cells/mL) were starved for 24 h in FluoroBrite DMEM medium (Gibco Cat. A18967-01), free of fetal bovine serum and glutathione. Next, cells were incubated with 0.5% (v/v) methanol (MeOH; control), methanol extract (500 μg/mL), purified Fractions I, II, III, IV, or V (500 μg/mL), 0.5% (v/v) ethanol (EtOH; Antimycin control) or Antimycin A (30 μM), as final concentrations in the assays. Eighteen hours after treatment, the cells were incubated with 0.8 μM MTRed during 15 min and MTRed positive cells were quantified by flow cytometry using an excitation laser beam at 640 nm and emission detector at 660 nm. Based on the MTRed intensities of the cell, they were distributed in three different populations nominated as THP-1 cells with low, middle or high ΔΨm. Results were expressed as percentage of cells distributed in each ΔΨm level. The assays were done in four independent experiments.

### Sequencing, 16S rRNA Gene Reconstruction, and Phylogenetic Analysis

Sequencing of the strain SGH1 genome was done by Illumina MiSeq system. The Illumina Nextera XT DNA sample preparation kit was used for library preparation, and the sequencing was done with a MiSeq V3 600-cycle kit. The sequencing produced paired-end reads of 300 bp long. The A5-MiSeq assembler version 20150522 with default parameters was used for genome assembly. The assembler incorporated the steps for data cleaning, error correction, assembly, and quality control (Tritt et al., 2012). The genome was then annotated by using the Rapid Annotations using Subsystem Technology (RAST) server version 4.0 (Aziz et al., 2008). The resulted annotated 16S rRNA genes were aligned against non-redundant nucleotide collection by using BLAST. The phylogenetic tree was constructed by multiple sequence alignment of the strain SGH1 16S rRNA gene (NCBI accession number: MN410435) against the sequences of different groups of *Haloarchaea* Class that were retrieved from NCBI. Multiple sequence alignment was created by ClustalW in MEGA 7 (Kumar et al., 2016) using ClustalW(1.6) weight matrix with default parameters. The evolutionary history was inferred by Neighbor joining method, and the evolutionary distances were computed by using *p*-distance method with a bootstrap of 1000.

### Statistical Analysis

Statistical data analysis was done by ANOVA with Bonferroni correction (confidence level: 95%, *p* ≤ 0.05) or *t*-test (confidence level: 95%, *p* ≤ 0.05).

## Results

After 2–3 weeks, microbial growth was only observed in Z8-NK medium supplemented with 25% NaCl and 10 mM L-proline, previously inoculated with samples from scrapped halite nodules. The turbidity of these cultures was clearly increased during the incubation time. From these cultures, Petri dishes containing fresh Z8-NK medium were inoculated using serial dilutions, and orange-red pigmented colonies were observed in highest abundance after 12 days. Thus, an isolate was obtained from a colony and characterized.

### Morphology of SGH1

**FIGURE 1:**
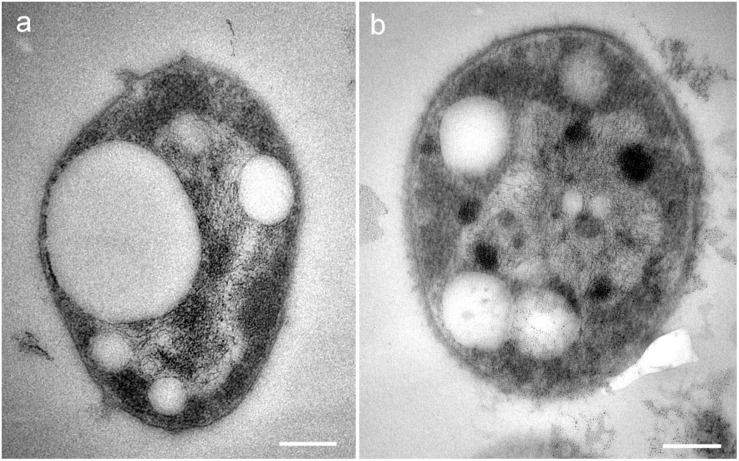
**(a,b)** TEM micrographs of isolate *Haloterrigena* sp. strain SGH1. Cells are unicellular cocci with cytoplasmic electron-lucent globular structures resembling gas vacuoles. Bar: 0.2 μm.

Strain SGH1 grown in solid Z8-MOD medium rendered convex, orange-red colonies with well-defined borders. Under the light microscope, strain SGH1 only occurred as one type of a Gram-negative, non-motile coccoid microorganism with an average diameter of 1–2 μm. Observation of the ultrastructure of the archaeon strain SGH1 by TEM showed a cytoplasm with several cytoplasmic globules that resembled the gas vesicles previously described in halophilic archaea (Oren, 2013) (Figures 1a,b).

### Phylogeny of SGH1

**FIGURE 2:**
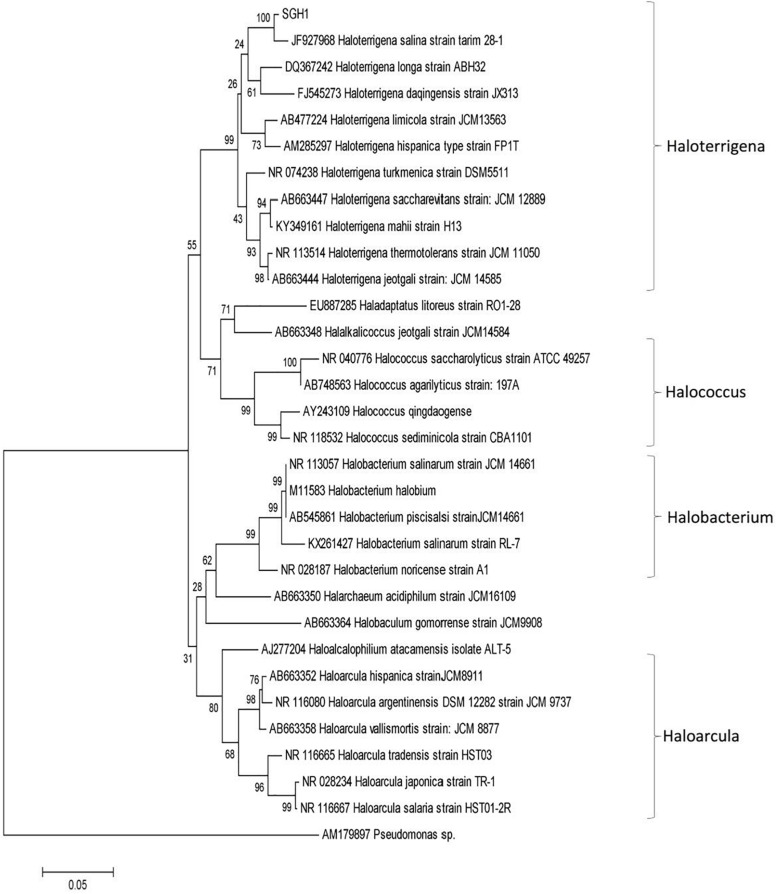
Phylogenetic analysis of isolate *Haloterrigena* sp. strain SGH1 by Neighbor joining method based on 16S rRNA gene sequences. The horizontal bar at the base of the figure represents 0.05 substitutions per nucleotide site. The percentage of trees in which the associated taxa clustered together is shown next to the branches, using a bootstrap of 1000. Evolutionary analysis was conducted in MEGA7.

The alignment of strain SGH1 16S rRNA gene sequence (NCBI accession number: MN410435) against NCBI non-redundant nucleotide collection showed a 99% of identity to *Haloterrigena turkmenica* (Saunders et al., 2010). The phylogenetic tree showed a close relationship with the *Haloterrigena* genera and specifically with *H. turkmenica* and *Haloterrigena salina* (Figure 2) (Gutiérrez et al., 2008; Saunders et al., 2010). Thus, strain SGH1 has been preliminarily denominated *Haloterrigena* sp. strain SGH1.

### Effect of Salinity, pH, and Temperature on the Growth of *Haloterrigena* sp. SGH1

Optimal growth for strain SGH1 was observed at 25% (w/v) NaCl at 45°C (Table 1). Strain SGH1 is also a neutrophile microorganism with optimal growth at pH 7–8 (data not shown). In addition, 15% (w/v) NaCl was the minimum salinity for strain SGH1 growth which was severely restricted at lower salinities.

**TABLE 1:** Effect of temperature and salinity on the specific growth rate (μ) of *Haloterrigena* sp. strain SGH1 adapted to grow at three NaCl concentrations.

NaCl (% w/v)	μ (h^–1^)		
	
	25°C	35°C	45°C
15	0.036 ± 0.003 (6)^a^	0.036 ± 0.002 (7)	0.048 ± 0.002 (9)^c, d^
25	0.029 ± 0.001 (6)	0.045 ± 0.001 (6)^b^	0.061 ± 0.002 (9)^c^
30	0.022 ± 0.001 (7)^a^	0.030 ± 0.003 (10)^b^	0.058 ± 0.001 (9)^d^

*The results are shown as mean ± standard error with n values in parentheses. Superscripts show significant differences (*p* ≤ 0.05).*

### Tolerance of *Haloterrigena* sp. Strain SGH1 to Magnesium and Perchlorate Salts

The effect of magnesium chloride (50 to 500 mM) and sodium or magnesium perchlorate (50 to 300 mM) on SGH1 aerobic growth was considered as a measure of tolerance of strain SGH1 for these salts, and results are shown in Table 2. The specific growth rate of strain SGH1 was not severely affected by the addition of the magnesium chloride and its growth rate decreased approximately 25% at the highest concentration used (0.5M MgCl_2_ x 6 H_2_O). SGH1 cells were also able to grow at 150 mM sodium or magnesium perchlorate with a decrease in its specific growth rate close to 40% (Table 2). Higher perchlorate concentrations were detrimental.

**TABLE 2:** Effect of magnesium and perchlorate on growth of *Haloterrigena* sp. strain SGH1.

Salt (mM)	Specific growth rate, μ (h^–1^)						
	
	0	50	100	150	300	400	500
MgCl_2_ x 6 H_2_O	0.029 ± 0.001	0.024 ± 0.002	0.018 ± 0.004	0.022 ± 0.0013	0.022 ± 0.0004	0.019 ± 0.0032	0.022 ± 0.0053
Mg(ClO_4_)_2_	0.029 ± 0.001	0.022 ± 0.0048	0.018 ± 0.004	0.017 ± 0.003	0.003 ± 0.004	0.0 ± 0.008	0.0 ± 0.001
NaClO_4_	0.029 ± 0.001	0.017 ± 0.001	0.017 ± 0,001	0.017 ± 0.0019	0.001 ± 0.0013	nd	nd

*Tolerance of strain SGH1 to MgCl_2_ x 6H_2_O (50 to 500 mM), to sodium perchlorate or magnesium perchlorate (50 to 300 mM) was investigated in medium Z8-MOD in the absence or additions of each salt. The specific growth rate for each condition was measured after 96 h of growth. Results shown as mean ± standard deviation (*n* = 3). nd: not done.*

### Bacterioruberin (BR) Content in *Haloterrigena* sp. Strain SGH1

SGH1 accumulated nearly 400 μg of BR per gram of dry biomass at late exponential phase of growth at 25–30% NaCl and 25–35°C (Table 3). Strain SGH1 can adapt to grow at 15% NaCl but its carotenoid content is severely restricted, rendering cultures with unpigmented cells. This depigmentation is a reversible and salt-dependent process since the carotenoid content can be recovered after transference and growth at higher salinity (Supplementary Figures S1–S3).

**TABLE 3:** Effect of salinity and temperature on the content of total carotenoids in *Haloterrigena* sp. strain SGH1.

NaCl (% w/v)	Carotenoids (μg/g dry biomass)		
	
	25°C	35°C	45°C
15	60.6 ± 4.62 (14)	19.8 ± 2.55 (31)	17.7 ± 2.08 (8)
25	437.5 ± 8.72 (16)^a^	400.9 ± 11.38 (23)^b^	256.4 ± 7.75 (12)^a,b,c^
30	416.6 ± 14.30 (17)^d^	394.4 ± 12.31 (24)^e^	319.6 ± 6.48 (11)^c,d,e^

*Results are shown as mean ± standard error with n values in parentheses. Superscripts show significant differences (*p* ≤ 0.05).*

### Identification of BR in *Haloterrigena* sp. Strain SGH1

**FIGURE 3:**
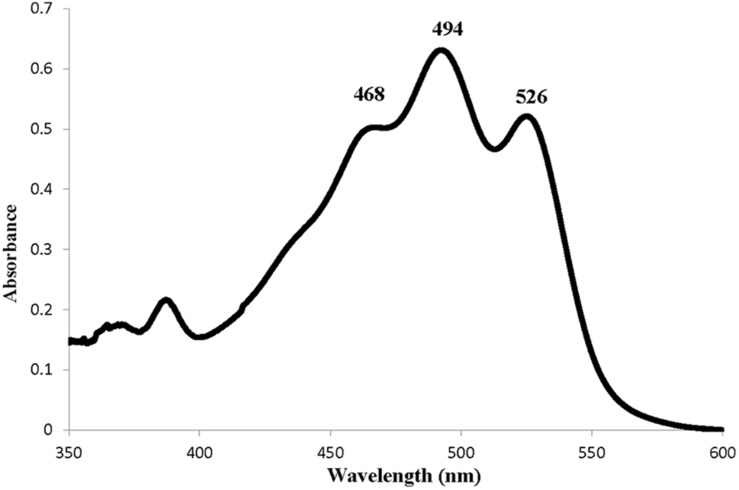
Absorption spectrum of a methanolic extract from *Haloterrigena* sp. strain SGH1 grown at 25% NaCl and 35°C.

The identification and composition of C-50 carotenoids in strain *Haloterrigena* sp. SGH1 was based on the data analyses of their chromatographic migrations, UV-Vis spectra, spectral fine structure and MS fragmentation patterns (Fong et al., 2001; Mandelli et al., 2012; De la Vega et al., 2016). The absorbance spectrum of the methanol extract of strain SGH1 showed the photometric fingerprint or three-fingered maxima profile that include maxima at 468, 494, and 526 nm (Figure 3), as it has been previously reported in archaea (Mandelli et al., 2012; Lorantfy et al., 2014; De la Vega et al., 2016). This was a preliminary evidence for the presence of BR-type molecules in SGH1 and the presence of a BR-type molecule in strain SGH1 cells was confirmed by Raman spectroscopy (Table 4) which showed three major Raman displacement signals (1.502, 1.147, and 995 cm^–1^) that were in close proximity with those informed for extracts from other haloarchaea (Jehlička et al., 2014; Dina et al., 2017).

**TABLE 4:** Raman displacement signals for *Haloterrigena* sp. strain SGH and other bacterioruberin-containing halophilic microorganisms.

Archaea	Raman displacement signal (cm^–1^)			References
*Haloterrigena* sp. strain SGH1	1,502	1,147	995	This work
*Halobacterium* sp. strain NRC-1	1,505	1,152	1,000	Marshall et al., 2007
*Rubrobacter radiotolerans* DSM 5868T	1,503	1,150	1,000	Imperi et al., 2007
*Halococcus dombrowskii* DSM14522T	1,507	1,152	1,002	Kaczor et al., 2011
*Halorubrum sodomense* ATCC 33755T	1,506	1,152	1,001	Jehlička and Oren, 2013
*Haloarcula vallismortis* ATCC 29715T	1,506	1,151	1,000	Jehlička et al., 2014
*Haloferax sulfurifontis* BL16 *Halorubrum sodomense* BL11 *Halobacterium salinarum* OL1	1,505	1,150	1,000	Dina et al., 2017

**FIGURE 4:**
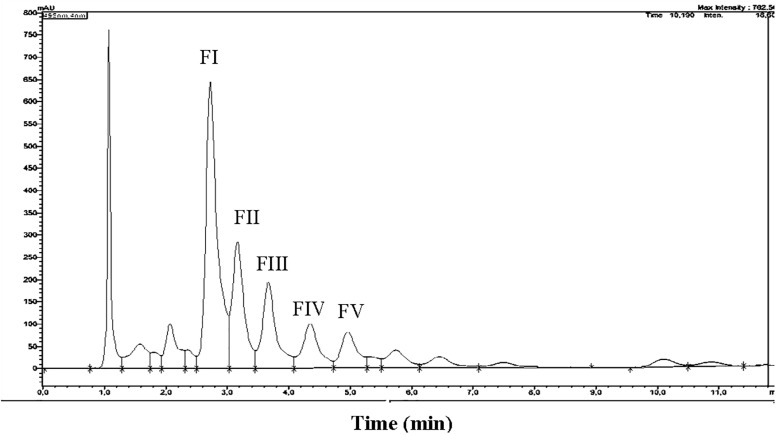
HPLC chromatogram of a methanolic extract from isolate *Haloterrigena* sp. strain SGH1, grown in Z8-MOD medium at 25% NaCl and 35°C. Cells were harvested, lyophilized and extracted with 100% methanol. An aliquot (50 μl) was injected in a HPLC Shimatzu Hitachi LC-20A Prominence, equipped with a reverse phase C18 column) and eluted with methanol:acetonitrile:water (85:10:5, v/v) at a flow rate of 1.0 ml/min, during 12 min. Five major fractions are observed and denominated FI to FV. Asterisks show the retention times used to compute the area of each fraction to obtain their relative abundance. Fraction collection was done at specific retention time avoiding the contamination between peaks; e.g., Fractions FI and FII were collected between the retention times of 2.687 to 3.098 and 3.17 to 3.45 min, respectively.

Total carotenoids from the methanol extracts of strain SGH1 were resolved into 17 fractions by HPLC/UHPLC. Nine fractions were separated by HPLC (Figure 4), being Fraction FI the most abundant. Further work was focused on the five major fractions (FI to FV) with retention times of 2.6, 3.1, 3.6, 4.2, and 4.9 min, respectively. All five fractions were recovered from the HPLC system and each one was injected in a UHPLC-MS system in order to separate subfractions and to obtain their retention times, UV-Vis absorption spectra and MS profiles.

**FIGURE 5:**
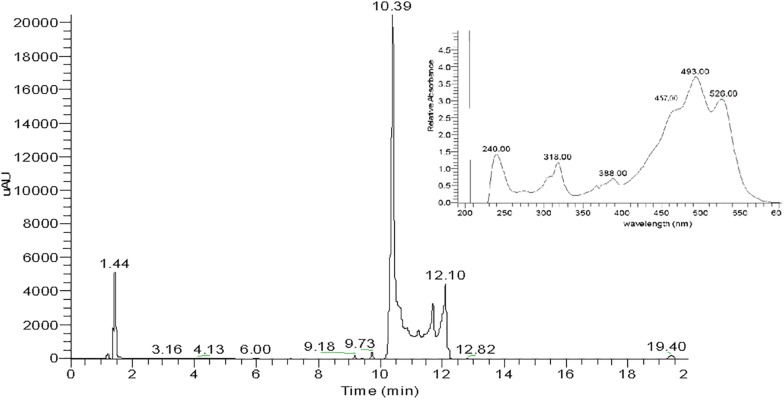
UHPLC chromatogram of Fraction FI from a methanolic extract of isolate *Haloterrigena* sp. strain SGH1. Lyophilized cells grown in Z8-MOD medium at 25% NaCl and 35°C were extracted with 100% methanol and Fraction FI was recovered after separation by HPLC. An aliquot of 10 μL of Fraction FI was injected in a UHPLC Thermo Scientific Dionex Ultimate-3000 system, equipped with a C18 column and eluted with a gradient of methanol: acetonitrile. The inset shows the UV-Vis spectrum of Subfraction FI-1 (retention time: 10.39 min), the most abundant UHPLC signal.

Fraction I was resolved into three signals after UHPLC, being Subfraction FI-1 the highest in intensity (Figure 5). The UV-Vis absorption spectrum of subfraction FI-1 showed the photometric fingerprint associated to BR-type molecules (457, 494, and 526 nm) and contained absorption maxima at the UV region (240, 318, and 388 nm) (Figure 5). The UHPLC system separated fractions FII FIII, FIV, and FV into 4, 4, 3, and 3 subfractions, respectively; the corresponding UHPLC chromatography and absorption spectra are shown in Supplementary Figure S4. All subfractions showed UV-Vis absorption spectra with photometric fingerprints associated to BR-like molecules and major differences in absorbance intensities were observed at their UV and visible absorption maxima (Figure 5 and Supplementary Figure S4). The absorbance maxima at 487–494 nm (peak II), 520–527 nm (peak III) and 388 nm (*cis*-peak or DII) were used to discriminate among geometric isomers and anhydrous derivatives of BR in strain *Haloterrigena* sp. SGH1 (absorbance ratios%III/II and%DII/II) (Fong et al., 2001; Meléndez-Martínez et al., 2006).

**FIGURE 6:**
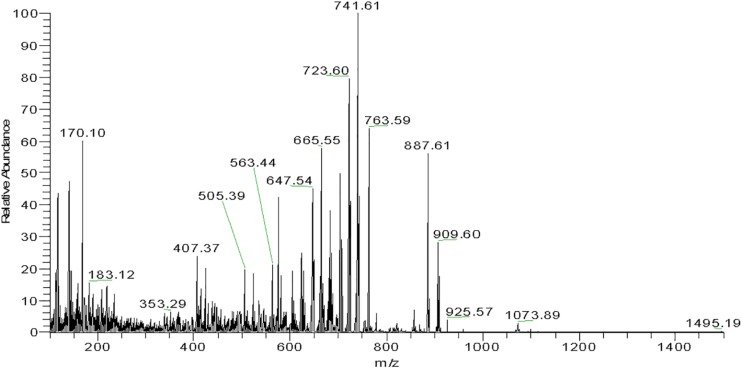
Mass spectrogram of subfraction FI.1 from *Haloterrigena* sp. SGH1. A family of fragments from subfraction FI.1 was obtained with a high-resolution mass spectrometer (Thermo, Bremen, Germany) coupled to a UHPLC Thermo Scientific Dionex Ultimate-3000 system. The molecular ion of 741.6 m/z is observed as the fragment with higher relative abundance.

Mass spectrometry provided the corresponding profiles for positively ionized fragments and their m/z for each major subfraction as shown in Figure 6 and Supplementary Figure S4, Supplementary Table S1. From the analysis of these spectroscopic and spectrophotometric data and comparison with previously reported information (Mandelli et al., 2012; Dina et al., 2017; Squillaci et al., 2017), it was inferred that *Haloterrigena* sp. strain SGH1 cells contain six C-50 molecules related to the BR family: four geometric isomers (5-*cis*-BR, 9-*cis*-BR, 13-*cis*-BR, and all-*trans*-BR) and two dehydrated derivatives (all-*trans*-tetra-anhydrous BR and *cis*-tetra-anhydrous BR) and, one the metabolic intermediate (all-*trans*-mono-anhydrous BR). Also, all-*trans*-BR was the major BR isomer accounting for nearly 65% of all BR-type carotenoids in SGH1. Fractions FI and FII are enriched in *all*-*trans*-BR and 5-*cis*-BR, respectively, and 9-*cis*-BR is the major carotenoid in FIII. Carotenoid composition plus UHPLC-MS and spectrophotometric data are summarized in Supplementary Table S1.

### Antioxidant Capacity of *Haloterrigena* sp. SGH1 Carotenoids

**FIGURE 7:**
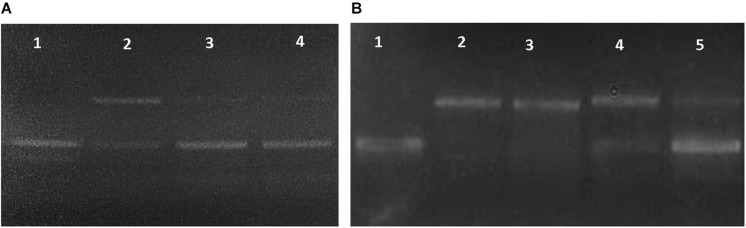
Dose-dependent DNA relaxation assay used to evaluate the antioxidant activity of the methanolic extract from *Haloterrigena* sp. SGH1. **(A,B)** Changes on the electrophoretic mobility of plasmid pUC19 were observed on 0.8% agarose gels after incubating the plasmid with water (as negative control: A1 and B1), Fenton reagent (as positive control: A2 and B2), Fenton reagent plus 0.001 μg/μL SGH1 extract (A3), Fenton reagent plus 0.002 μg/μL SGH1 extract (A4 and B5), Fenton reagent plus 0.1 μg/μL β-carotene (B3) and, Fenton reagent plus 0.1 μg/μL Trolox (B4).

Three different assays were used to evaluate the antioxidant capacity of BR from *Haloterrigena* sp. SGH1. First, the protective role of SGH1 extracts against oxidative damage of DNA was tested using plasmid pUC19. Incubation of plasmid DNA with the Fenton’s reagent rendered a relaxation of the original supercoiled DNA with a decreased electrophoretic mobility (Figure 7, lanes A2 and B2). DNA relaxation was clearly diminished by the addition of SGH1 extract in the assay; protection to DNA oxidation increasing from 50 to 70% when the concentration of the extract was doubled (Figure 7, lanes A3 and A4). Neither Trolox nor β-carotene was as effective antioxidant as the methanolic extract from SGH1 which was tested at a 50–100-fold lower concentration (Figure 7). Second, the ABTS assay showed that methanolic extracts of SGH1 were highly efficient in scavenging free radicals; the ABTS results, expressed as TEAC (Trolox equivalent antioxidant capacity), indicated that the antioxidant activity of SGH1 carotenoids was nearly 8, 9, and 26 times higher than Trolox, beta-carotene or astaxanthin, respectively. Also, the IC_50_ for SGH1 extracts was nearly five times lower than Trolox or beta-carotene and 19-fold lower than astaxanthin (Table 5). Also, the ABTS/TEAC values for the HPLC fractions FI, FII, FIII, and extract SGH1 showed a close similarity. Third, the TEAC values obtained by the FRAP assay indicated that SGH1 carotenoids had an antioxidant capacity nearly 10, 18 and 20 times higher than Trolox, beta-carotene or astaxanthin, respectively. The TEAC values for the HPLC fraction FI and the SGH1 extract were comparable but lower than those for Fractions FII and FIII (Table 5).

**TABLE 5:** Antioxidant activity of bacterioruberin from isolate *Haloterrigena* sp. strain SGH1, Trolox, and C_40_ carotenoids.

Antioxidant	TEAC (ABTS)		TEAC (FRAP)
		
	μg/ml	IC_50_	μg/ml
Trolox	1.0	3.7	1.0
Beta-carotene	0.9 ± 0.4 (80)	4.2	0.6 ± 0.3 (40)
Astaxanthin	0.3 ± 0.0 (12)	15.1	0.5 ± 0.1 (12)
SGH1 (extract)	7.7 ± 3.8 (70)	0.8	10.5 ± 3.3 (82)
Fraction I	6.8 ± 2.0	1.0	7.8 ± 2.1
Fraction II	7.8 ± 2.8	0.4	27.5 ± 5.8
Fraction III	8.7 ± 3.0	0.4	16.7 ± 5.6

*The results are shown as mean ± standard deviation with n values in parentheses.*

### Cellular Toxicity of Methanol Extracts and Carotenoids From Strain SGH1

**FIGURE 8:**
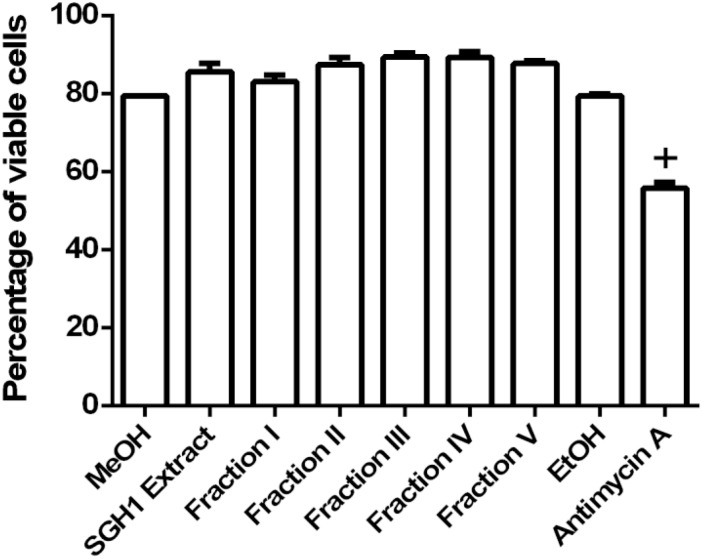
Toxicity of carotenoids from isolate *Haloterrigena* sp. strain SGH1 against THP-1 cells. The methanolic extract (SGH1 Extract) and purified fractions F-I, F-II, F-III, F-IV and F-V from *Haloterrigena* sp. strain SGH1 (500 μg/mL of each as final concentrations in the assays), were incubated with THP-1 cells for 18 hours. After that, cells were incubated with propidium iodide (PI) by 15 min and viable cells (PI negative) were quantified by flow cytometry. Methanol (MeOH) and Ethanol (EtOH) were used as negative control and 30 μM Antimycin A was used as positive control. The results were expressed as percentage of viable cells, 18 h post-treatment. *T*-test: ^+^*p* < 0.5 compared with the control group (MeOH), *n* = 4.

Evaluation of THP-1 cell viability was conducted by the binding assay of the impermeant fluorogen PI to DNA. The percentage of viable THP-1 cells in the presence of methanol extracts from strain SGH1 or each purified fractions I, II, III, IV, or V were 85.52 ± 2.19, 83.08 ± 1.65, 87.44 ± 1.82, 89.37 ± 1.13, 89.28 ± 1.55, and 87.72 ± 0.75%, respectively, showing that they were not toxic to THP-1 cells. Viable cells dropped to 55.83 ± 1.49% in presence of non-lethal 50 μM Antimycin A and a 79.44 ± 0.00% survival was observed in the control cells incubated in methanol (Figure 8). The nearly 20% of dead cells observed in the control group in methanol is consequence of the 48-h starvation of THP-1 cells in FluoroBrite medium, in the absence of the antioxidant reduced glutathione.

**FIGURE 9:**
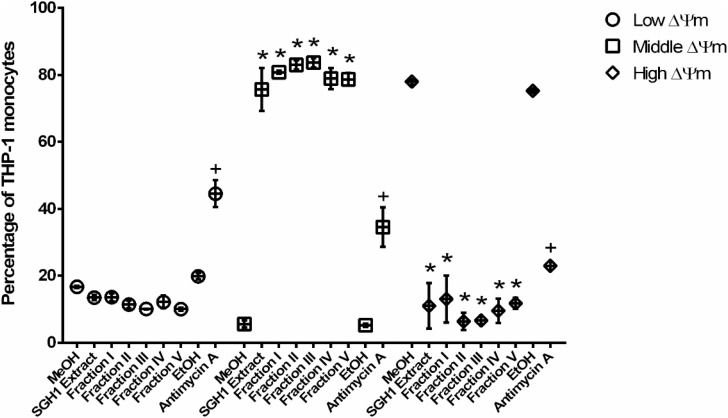
Cellular toxicity against THP-1 cells of carotenoids from isolate *Haloterrigena* sp. strain SGH1. The methanolic extract (SGH1 Extract) and purified fractions F-I, F-II, F-III, F-IV, or F-V from *Haloterrigena* sp. strain SGH1 (500 μg/mL of each as final concentrations in the assays), were incubated with THP-1 cells for 12 hours. After that, cells were incubated with MitoStatus Red (MTRed) by 15 min and percentage of cells with different intensities of fluorescence were quantified by flow cytometry. Methanol (MeOH) and Ethanol (EtOH) was used as negative control. Antimycin A (30 μM) was used as positive control. Graphic represents percentage (mean ± SEM) of THP-1 monocytes distributed among three different fluorescence intensities and nominated cells with Low, Middle or High mitochondrial membrane potential (ΔΨm). T-test: * and ^+^*p* < 0.5 compared with the control group (MeOH or EtOH), *n* = 4.

Mitochondria membrane potential was assayed as a second criterium for BR toxicity. The fluorescence intensity measured in THP-1 cells incubated with the lipophilic, fluorescent MTRed, divided the cell population into three different groups with low, middle or high ΔΨm (Supplementary Figure S5). SGH1 methanolic extracts and purified fractions I, II, III, IV, and V decreased significantly the percentage of high-ΔΨm population from 78 ± 0.9% (MeOH) to 11 ± 6.8, 13 ± 7.0, 6.4 ± 2.5, 6.6 ± 1.1, 9.6 ± 3.6, and 11.7 ± 1.7%, respectively. Concomitantly, SGH1 methanolic extracts and purified fractions I, II, III, IV, and V increased significantly the percentage of middle-ΔΨm population from 5.6 ± 1.0% (MeOH) to 75.6 ± 6.4, 80.8 ± 0.5, 83.0 ± 1.4, 83.6 ± 1.4, 78.9 ± 3.1, and 78.7 ± 1.9%, respectively. Interestingly, SGH1 methanolic extracts and purified fractions I, II, III, IV, and V decreased the percentage of low-ΔΨm population from 16.7 ± 0.4% (MeOH) to 13.5 ± 0.8, 13.6 ± 1.2, 11.4 ± 1.1, 10.1 ± 0.1, 12.2 ± 1.9, and 10.0 ± 0.5%, respectively. Antimycin A, an inhibitor of mitochondrial electron transport (Complex III) decreased the percentage of high-ΔΨm population from 75.2 ± 0.7% (EtOH) to 23.0 ± 1.3% and increased the percentage of middle-ΔΨm and low-ΔΨm population from 5.2 ± 0.5 to 34.5 ± 5.9% and from 19.8 ± 1.0 to 44.5 ± 4.0%, respectively, as it was expected (Figure 9).

## Discussion

Extreme environments on Earth impose physical and chemical limits to life forms (Merino et al., 2019). The hyperarid core of the Atacama Desert has been subjected to high desiccation and solar radiation for millions of years and it is considered a Mars analog (Navarro-González et al., 2003; Clarke, 2006). Desiccation and high solar radiation are the two major environmental factors that limit abundance and diversity of microbial life in Atacama (Korehi et al., 2013; Wierzchos et al., 2015; Gómez-Silva, 2018; Meslier et al., 2018; Schulze-Makush et al., 2018). Atacama is a poly-extreme environment previously known as a mineral-rich but sterile territory. However, advances on microbiology during the last two decades have demonstrated the presence of an unexpected microbial diversity although limited in abundance (Warren-Rhodes et al., 2006; de los Ríos et al., 2010; Gómez-Silva, 2010, 2018; Robinson et al., 2015; Bull et al., 2016; Crits-Christoph et al., 2016a, b; Meslier et al., 2018; Wierzchos et al., 2018).

Microbiological studies in Atacama have also provided new insights on the lithobiontic life organized as biofilms inhabiting inside or on the surface of rock substrates. Lithic habitats provide physical and chemical conditions needed for microbial life to cope with high solar radiation and desiccation and prompted the notion that lithic niches would be the last solution for the survival of microbial life under hyperarid environments (Wierzchos et al., 2006; Davila et al., 2008; Robinson et al., 2015; Gómez-Silva, 2018; Meslier et al., 2018; Wierzchos et al., 2018; Uritskiy et al., 2019).

### Phylogenetic Relationships of Strain SGH1

Halite nodules in Salar Grande, near the eastern slopes of the Coastal Range in northern Chile, are colonized by a complex microbial community of unculturable members of the three domains of life and viruses (Gómez-Silva, 2010, 2018; Robinson et al., 2015; Crits-Christoph et al., 2016a, b; Santiago et al., 2018; Uritskiy et al., 2019). Metagenomic analyses of DNA from halites and soil samples at Salar Grande showed the presence of members related taxonomically to the archaeal family *Halobacteriaceae* and the genera *Haloterrigena*, *Halococcus*, and *Halorhabdus* (Robinson et al., 2015). Strain SGH1 was isolated from endolithic biofilms confined within halites at Salar Grande. Sequence analysis of the 16S rRNA gene showed that strain SGH1 belongs to the *Haloterrigena* genus with a close phylogenetic relationship (99% identity) to *H. turkmenica* 4k^T^ (Saunders et al., 2010) and *H. salina* (Gutiérrez et al., 2008). *H. turkmenica* 4k^T^ is an archaeon isolated from sulfated soils in Turkmenistan and described as the type species for the genus *Haloterrigena* (Saunders et al., 2010) whereas *H*. *salina* is an extreme halophilic archaeon isolated from a saline lake in China. Thus, the first culturable microorganism from endolithic communities colonizing halites in Atacama has been denominated *Haloterrigena* sp. strain SGH1 and further work is needed to assign its corresponding species.

The positive effect of KCl to the growth medium was indicative that *Haloterrigena* sp. strain SGH1 would use the salt-in strategy to cope with high salinities, as shown for other extreme halophiles (Oren, 1999; Strahl and Greie, 2008). Optimal growth under these conditions resulted in a doubling time of 16 h. Strain SGH1 can grow at high salinity (15 to 30%, w/v NaCl), but growth was severely restricted at lower salinities, demonstrating extreme halophilism. Optimal salinity and temperature for SGH1 growth were like those reported for the haloarchaeon *Halorubrum* sp. SH1 (De la Vega et al., 2016). Comparatively, the type species *H. turkmenica* 4k^T^, described as a rod-shaped archaeon, is considered to be the fastest growing member of the *Halobacteriaceae*, with a generation time of 1.5 h at 51°C and able to growth at least at 2% (w/v) NaCl (Saunders et al., 2010), which is an ampler salinity range compared with the more restrictive salinity limits shown by *Haloterrigena* sp. strain SGH1.

### Bacterioruberin Biosynthesis

BR synthesis in *Haloterrigena* sp. strain SGH1 is a reversible, salt-dependent metabolic process and the maximum carotenoid content was obtained in cells grown at 25°C and 25% NaCl. Comparatively, a lower carotenoid content has been registered for other halophilic archaea; e.g., 45 μg g^–1^ of dry biomass in *Halococcus morrhuae* (Mandelli et al., 2012), 75 μg g^–1^ of dry biomass in *H. turkmenica* (Squillaci et al., 2017) and 335 μg g^–1^ of dry biomass *Haloarcula japonica* (Yatsunami et al., 2014). Recently, Zalazar et al. (2019) have reported that the bacterioruberin content (220 mg g^–1^ dry weight) in a hyperpigmented, genetically modified *Haloferax volcanii* strain HVLON3 is higher than in other haloarchaea. The red-pigmented strain SGH1 accumulates BR when growing at high salinity (25% NaCl) but the BR content decreased nearly 20-fold after the SGH1 cells are transferred and adapted to grow at low salinity (15% NaCl). Depigmentation is a reversible process in which the BR biosynthesis resumed after the unpigmented cells are transferred and grown at high salinity, reaching the maximum BR content observed in SGH1 cells adapted to grow at high salinity. Thus, BR content correlates reversibly with the salinity of the growth medium, an adaptive process not previously reported in other *Haloterrigena* species. Several studies on the reversible effect of salinity on carotenoid content are available; for example, (i) two extreme halophilic archaea, *Halobacterium cutirubrum* and *Halobacterium mediterranei*, showed that their C_40_ and C_50_ carotenoids content was also salt-modulated in an opposite fashion, that is, carotenoids in *H*. *mediterranei* decreased severely at high salinity, while high carotenoid content was observed when *H*. *cutirubrum* were grown at 20–35% salinity (Kates, 1986); (ii) Kushwaha et al. (1982) reported that total carotenoid content increased 18–19-fold when the extreme halophilic strain R-4, isolated from salt ponds in Spain, was grown at low salinity; (iii) the halophilic archaea *Haloferax mediterranei*, *Haloferax volcanii*, *Halobacterium* strain SP-2, *Halorubrum* strain SP-4 and, *Haloferax alexandrinus* strain TM^T^ showed an increase on carotenoid content at low salinity, probably as a lysis avoidance mechanism (Calegari-Santos et al., 2016) and, (iv) the moderately halophilic archaeon *Haloferax volcanii* transferred from low (12.5% NaCl) to high salinity (20% NaCl) showed a twofold increment on the expression and a threefold increase in specific activity of HMG-CoA reductase, an enzyme involved in isoprenoide biosynthesis leading to carotenoids in haloarchaea, but a 1.5-fold decrease in carotenoid content (Bidle et al., 2007). In addition, the yeast *Rhodotorula mucilaginosa* also showed a reversible naftifine-dependent carotenoid depigmentation process (Mot et al., 2017). Considering that BR molecules play a role in cell photoprotection and are efficient radical scavengers (Shahmohammadi et al., 1998) and, strain SGH1 does not grow at salinities below 15% NaCl, we propose that accumulation of BR at high salinity would reinforce cell membranes, increasing hydrophobicity and minimizing intracellular water loss in *Haloterrigena* sp. strain SGH1 as well as in other extremely halophilic archaea with similar salt-dependent control on their carotenoid biosynthesis. We also propose that the reversible salt-dependent adaptive process for BR biosynthesis in strain SGH1 may be a novel regulatory mechanism in which the intracellular concentration of potassium affects the expression of genes or the activity of one or more key enzymes; for example, lycopene elongase, involved in BR synthesis.

Previously, the effect of nicotine on carotenoid biosynthesis was studied by Kushwaha and Kates (1976) in *H. cutirubrum*, showing that lycopene is the C_40_ precursor for C_50_ carotenoid formation. In addition, the use of mutants of *Haloarcula japonica* by Yang et al. (2015), allowed the elucidation of the biosynthetic pathway from lycopene to BR. Recently, the effect of nicotine on carotenoid metabolism on three species of *Halobacteria* has been revisited by Oren et al. (2018), using different analytical techniques and supporting the biosynthetic pathway elucidated for *Haloarcula japonica* by Yang et al. (2015) and providing evidence that nicotine inhibited the conversion of *bis*anhydrobacterioruberin to monoanhydrobacterioruberin, and the elongation of lycopene to a C_45_ intermediate.

### Carotenoid Composition in *Haloterrigena* sp. Strain SGH1

BR is a general term that includes a family of geometric isomers of polyene molecules in haloarchaea. BR is a lineal C_50_ polyene chain with a conjugated 13-double bonds system plus two hydroxyl groups at each extreme (Kushwaha et al., 1975; Yang et al., 2015). BR, as family of geometric isomers, is found in cells subjected to high solar radiation, salinity and desiccation, playing several biological roles such as protection to photo-oxidative damage, as a strong radical scavenger (Shahmohammadi et al., 1998; Gabani and Singh, 2013). BR-like molecules were the principal carotenoids accumulated by *Haloterrigena* sp. strain SGH1, based on the UV-Vis spectra (photometric fingerprint) and the three major Raman displacement signals (1502, 1147, and 995 cm^–1^) observed in the methanolic extracts (Imperi et al., 2007; Marshall et al., 2007; Kaczor et al., 2011; Mandelli et al., 2012; Jehlička and Oren, 2013; Jehlička et al., 2014; Dina et al., 2017; Squillaci et al., 2017). According to Jehlièka et al. (2019) and under green excitation laser, the strong Raman signals near 1506, 1152, and 1000 cm^–1^, are associated to C = C stretching band, to C–C stretching band and methyl groups in the polyene chain bound to C–C bonds, respectively. Those minor Raman displacement signals observed at 1,300–1,450 cm^–1^ could be associated to methyl group deformations and indicative of the presence of glycine betaine (Dina et al., 2017). Then, the osmolite glycine betaine may be preliminarily considered as a component of the osmoregulatory system in *Haloterrigena* sp. SGH1, acting complementary to KCl.

Methanolic extracts of isolate SGH1 were chromatographically separated into 17 fractions. Those fractions with the highest relative abundance were selected for further analyses. The identification of the carotenoids was based on their UV-Vis spectra, spectral fine structure and MS fragmentation patterns. We found that BR molecules from *Haloterrigena* sp. SGH1 included four geometric isomers (all-*trans*-BR, 5-*cis*-BR, 9-*cis*-BR, and 13-*cis*-BR), two dehydrated derivatives (all-*trans*-tetra-anhydrous-BR, *cis*-tetraanhydrous-BR) and one metabolic intermediate (*cis*-mono-anhydrous-BR). Among them, all-*trans*-BR was the most abundant carotenoid accounting for 65% of total carotenoids in strain SGH1 and all geometric isomers of BR represented nearly 80% of total carotenoids in SGH1. The presence of minor carotenoids (e.g., glycosylated BR) was not investigated. In addition, the use of a C18 column in the HPLC system showed limitations in the separation of fractions; for example, Supplementary Figure S4C shows one major peak at RT 11.21 min; this peak is also shown in Supplementary Figure S4D, between subfraction FIII.1 and FIII.2) but at a very low abundance when compared with Supplementary Figure S4C. Then, the peak at RT 11.24 min, between subfraction FIII.1 and FIII.2, can be identified as 5*-cis*-BR. In a similar way, the major signal in Fig. FIII.2 has a RT of 11.71 min, and it was identified as 9-*cis*-BR in Supplementary Table S1. This same peak was observed in Supplementary Figures S4F,G with a RT of 11.72 min, located between peaks FIV.1 and FIV.2, with also a minor abundance in comparison with peak FIII.2.

Different C_50_ carotenoids have been reported in other halophilic archaea, as expected for studies carried out with different genera and species. Abundant C_50_ carotenoids and minor contents of C_30_, C_40_, and C_51_ carotenoids were found in *H. turkmenica* DMS-5511 (Squillaci et al., 2017). Extracts from *H. turkmenica* were resolved into 32 HPLC signals and 21 of these peaks were identified as molecules belonging to the BR family by MS, collision induced dissociation and UV-Vis spectra. These molecules included all-*trans*-BR (65% of total carotenoids), five different *cis*-isomers of BR and 10 different dehydrated isomer derivatives of BR, and all-*trans*-BR (Squillaci et al., 2017). The presence and absorbance intensity at the UV spectral region shown by BR molecules is indicative of a differential role they may play in the cellular protection to UV photons.

All-*trans* BR isomers have a linear arrangement of its doble bonds but *cis*-BR isomers are bended molecules (Fong et al., 2001). According to Mandelli et al. (2012), *cis*-isomers have higher absorbance than *trans*-isomers at the *cis*-peak (388 nm) which becomes more intense as the *cis* double bond gets closer to the center of the molecules (Meléndez-Martínez et al., 2006). The relatively low absorbance of subfraction FI-1 at 388 nm supported the identification of the high-content of all-*trans*-BR molecules in *Haloterrigena* sp. strain SGH1. Different composition of BR molecules was also reported for *Haloarcula japonica*, *Halorubrum* sp. SH1 and *Haloferax volcanii*, showing the presence of geometric isomers and dehydrated derivatives of BR (Mandelli et al., 2012; Yatsunami et al., 2014; De la Vega et al., 2016). Compared with others reports, differences in the analytical systems, used in this work (e.g., the use of chromatographic columns C18 or C30), and extraction methods, including the solvents, could explain the apparently lower number of BR isomers and derivatives found in *Haloterrigena* sp. strain SGH1 (Mandelli et al., 2012; Lorantfy et al., 2014; Yatsunami et al., 2014; De la Vega et al., 2016; Squillaci et al., 2017).

### Tolerance of Strain SGH1 to Perchlorate and Magnesium Salts

Lithobiontic microbial consortia thriving within halite pinnacles from Salar Grande are in a ‘foggy environment’ (Cereceda et al., 2008). These environmental conditions suggest that water is condensing frequently on the halite surfaces by absorption of fog micro-droplets. Moreover, liquid brines can be formed within halites by sodium chloride deliquescence at a minimum deliquescence relative humidity (RH) of 75% (Davila et al., 2008) and by capillary condensation when RH is above 55% (Wierzchos et al., 2012). These conditions lead to the presence of almost constant moisture in these salt pinnacles (Robinson et al., 2015). Chloride and perchlorate salts form brines and hydrates at a lower relative humidity level (Gu et al., 2017; Jia et al., 2018). Perchlorate salts are present in Mars soils as well as at the Atacama Desert, and microbial growth in the presence of these salts has important metabolic and astrobiological implications (Jackson et al., 2015; Gu et al., 2017; Jia et al., 2018; Laye and DasSarma, 2018; Vega et al., 2018; Montgomery et al., 2019; Nazarious et al., 2019). Perchlorate reduction has been well-studied in Bacteria representatives, but perchlorate metabolism has expanded to the Archaea domain after studies on *Archaeoglobus fulgidus* strains and other haloarchaea (Liebensteiner et al., 2013; Oren et al., 2014). The halophilic archaeon *Halorubrum lacusprofundi* could grow aerobically at half of its growth rate in 0.3M NaClO_4_ or 0.1M Mg perchlorate; growth under anaerobiosis was limited to a maximum of 40 mM perchlorate salts (Laye and DasSarma, 2018). Similar results were reported for several members of the family *Halobacteriaceae* which were resistant to perchlorate when growing in a NaCl-based medium (Oren et al., 2014). Evidence of perchlorate at saline soils in Yungay, at the hyperarid core of Atacama, and its oxidative effects on organic matter, has been recently reported by Montgomery et al. (2019); then, tolerance to perchlorate was investigated in strain SGH1, considering that it is the first extremely halophilic archaeon isolated from halite communities. *Haloterrigena* sp. strain SGH1 could grow aerobically in 25% (w/v) NaCl at a maximal concentration of 150 mM magnesium or sodium perchlorate, with a 40% drop in its specific growth rate, suggesting the existence and expression of SGH1 genes involved in perchlorate tolerance.

Although Mg is essential for life, high concentrations of magnesium chloride are considered a serious stressor that limits microbial growth, mostly acting as a chaotropic agent affecting the structure and activity of macromolecules. Halophilic archaea that can sustain growth at molar concentrations of magnesium chloride do so in the presence of kosmotrope solutes such as high NaCl concentrations (Hallsworth et al., 2007). Tolerance of *Haloterrigena* sp. strain SGH1 to magnesium was investigated by adding increasing concentrations of MgCl_2_ until it was equivalent to a 2,500-fold increment from 0.2 mM MgCl_2_, the concentration in the growth medium. A drop of 25% on its growth rate was observed when isolate SGH1 was grown in 25% (w/v) NaCl plus 0.5M magnesium chloride. The magnesium concentration within the endolithic colonization zone in halites from Salar Grande is 0.4 mg kg^–1^ nearly 10 times lower than the concentration in the growth medium. Thus, isolate SGH1 is tolerant to chaotropic agents such as high magnesium concentrations during growth in a medium at high salinity (25% NaCl). This agrees with growth at a maximal concentration of 0.42M MgCl_2_ and 2.76M NaCl, of halophilic isolates from a MgCl_2_-dominated hypersaline lake at the Mediterranean Sea (Hallsworth et al., 2007). The tolerance to high concentrations of magnesium chloride and perchlorate salts by isolate SGH1 provide us with a glimpse on the metabolic capabilities of this extreme halophile from Atacama, opening potential applications on the biological removal of perchlorates from nitrate-rich fertilizers and natural waters.

### Antioxidant Capabilities of *Haloterrigena* sp. SGH1 Carotenoids

Methanolic extracts from *Haloterrigena* sp. strain SGH1 are a source of protective molecules that diminished damage to DNA from oxidative stressors (Shahmohammadi et al., 1998). Also, the methanolic extracts, as well as the SGH1 fractions purified chromatographically by HPLC, showed higher antioxidant power than well-known radical scavengers such as Trolox or C_40_ carotenoids. The antioxidant activities of fractions FI and FII (enriched in all-*trans*-BR and 5-*cis*-BR, respectively) were similar or higher than their original methanolic extracts, depending upon de antioxidant assay used. This is the first report of antioxidant activity of purified isomers of BR. Our results are in clear correspondence with previous reports using extracts of halophilic archaea from various origins; for example, TEAC values for carotenoid extracts from two halophilic archaea, *H*. *morrhuae* and *Halobacterium salinarium*, were 5.1–5.3, with IC_50_ of 0.8 μg/mL (Mandelli et al., 2012). Also, carotenoid extracts from *H. turkmenica* DMS-5511 showed higher antioxidant activity than reference antioxidants such as alfa-tocopherol or ascorbic acid (Squillaci et al., 2017). Finally, seven halophilic archaeal strains whose C_50_ carotenoids BR and its derivatives mono-anhydrous-BR and *bis*-anhydrous-BR were predominant, had antioxidant capacities significantly higher than β-carotene as determined by 1,1-diphenyl-2-picrylhydrazyl radical scavenging assay (Hou and Cui, 2018).

### BR Cytotoxicity

We report here the effect of BR isomers and derivatives on human THP-1 monocytes. HP-1 cells are bloodstream-circulating, non-adherent cells and a good model to be exposed to high concentrations of novel compounds that eventually may be used in biomedical applications (Pick et al., 2004). All cells were subjected to a previous 24-h cells starvation in FluoroBrite DMEM media, which is free of fetal bovine serum and glutathione. Using an assay based on a membrane impermeant fluorogen, BR molecules from *Haloterrigena* sp. strain SGH1 increased the percentage of viable cells (90% with Fraction III, as the most effective) in comparison with control cells (80% viability). Then, BR isomers and derivatives are not toxic to cultured human cells. The addition of these antioxidant and non-toxic molecules improved the viability of starved THP-1 cells for at least 42 h (24 h of starving plus 18 h of treatment).

The methanolic extract and all BR fractions from SGH1 decreased the percentage of high ΔΨm-cells and Fractions II and Fraction III were the most effective ones (78 to 6 and 78 to 7%, respectively). A parallel increase in the percentage of middle ΔΨm-cells was observed, and Fractions II and Fraction III were most effective (6 to 83 and 6 to 84%, respectively). Neither the methanolic extract nor all the purified fractions increased the percentage of cells showing low-ΔΨm, in contrast to the effect of 30 μM Antimycin A (46% increase), which is used as a positive control for a partial disruption of ΔΨm by inhibiting Complex III at the mitochondrial electron transport chains. In consequence, BR molecules from *Haloterrigena* sp. strain SGH1 decreased the mitochondrial membrane potential of THP-1 cells at a non-toxic level. The mechanism for this effect requires more investigation and we propose that the activation of mitochondrial uncoupling proteins may be involved. The existence of a feedback loop between ROS and the inner mitochondrial membrane proton leak has been proposed (Skulachev, 1996): ROS would increase proton leak which, in turn, would decrease ROS production in order to maintain mitochondrial electron transport (Skulachev, 1996). Since proton leak could compromise significantly the magnitude of ΔΨm (Brand et al., 1994; Brookes, 1998), we propose that BR molecules would scavenge ROS and interfere the feedback loop with an initial burst increase in ΔΨm followed by the activation of mitochondrial uncoupling proteins with a subsequent proton leak that would diminish the mitochondrial membrane potential.

Carotenoids from halophilic microorganisms have gained an increasing attention considering their potential in biotechnology and biomedicine (Rodrigo-Baños et al., 2015). Recently, a fraction from a semipreparative MPLC from a pigmented and UV-resistant Antarctic bacteria *Arthrobacter agilis* 50Cyt, containing all-*trans*-BR, was shown to be not phototoxic to cultured murine fibroblasts; however, the glycosylated BR molecules were highly phototoxic and cytotoxic to these cells (Silva et al., 2019). Also, cytotoxicity to HepG2 was demonstrated in cell extracts from a *Halobacterium halobium* and extracts from *H. salinarium* and *Haloferax volcanii* strains isolated at a solar saltern in Tunisia and brines in India (Abbes et al., 2013; Sikkandar et al., 2013). In fact, methanol extract of a genetically modified hyperpigmented *Haloferax volcanii* strain, rich in BR, showed beneficial effects on cryopreserved ram sperm, improving viability and motility after thawing (Zalazar et al., 2019). Our work has demonstrated that purified isomers and derivatives of BR from *Haloterrigena* sp. strain SGH1 are not cytotoxic to human monocytes. This and further studies will provide the basis for considering the potential use of purified BR carotenoids from *Haloterrigena* sp. strain SGH1 in biomedical applications or as an ingredient in edible products.

## Conclusion

Lithobiontic microbial consortia in Atacama halites form biofilms with a surprisingly complex diversity that includes unculturable members of the three domains of life and viruses. *Haloterrigena* sp. strain SGH1 is the first extreme halophilic archaeon isolated from endolithic microbial communities inhabiting halites at the Atacama Desert. BR content in SGH1 is one of the highest reported in halophilic archaea. BR biosynthesis in SGH1 cells is a reversible salinity-modulated process: cells grown at high salinity (25%) showed maximal BR content, but growth at low salinity (15%) rendered unpigmented cells. Growth of strain SGH1 at 25% (w/v) NaCl, was limited by magnesium chloride and sodium or magnesium perchlorate at maximal tolerable concentrations of 500 and 150 mM, respectively; thus, isolate SGH1 can be considered a new experimental model in which to study genes and enzymes associated to aerobic/anaerobic perchlorate metabolism and its applications on biological removal of perchlorates from nitrate-rich fertilizers and natural waters. Also, this is the first report that shows that the antioxidant activity of purified isomers of BR is higher than Trolox, beta-carotene, and astaxanthin. In addition, we report the first evaluation of BR toxicity on THP-1 cells concluding that purified BR isomers are not toxic to cultured human cells and open the opportunity for new applications in pharmaceutical, food and medical products.

## Data Availability Statement

Nucleotide sequence used in this work can be found in NCBI accession number MN410435.

## Author Contributions

NF conducted extraction, purification and identification of carotenoids and evaluated their biochemical properties. SH isolated and purifies strain SGH1. NF, MV, and AG implemented and evaluated the antioxidant activity of extracts and purified fractions. LZ, FF, and BP conducted cytotoxic assays. CS-A applied HPLC to extracts and collected fractions. CA, JW, and VS-E conducted TEM and JW the Raman studies. SH, AG, CV, and JA conducted genomic studies. RB-G analyzed results and reviewed critically the manuscript. BG-S conceived and designed the studies and wrote the manuscript. All authors read and approved the manuscript.

## Conflict of Interest

The authors declare that the research was conducted in the absence of any commercial or financial relationships that could be construed as a potential conflict of interest.

## Footnotes

1 https://imagej.net/ImageJ
