# Supplementary material for: Haloterrigena sp. Strain SGH1, a Bacterioruberin-Rich, Perchlorate-Tolerant Halophilic Archaeon Isolated From Halite Microbial Communities, Atacama Desert, Chile
Source: Front Microbiol. 2020 Mar 5;11:324. doi: 10.3389/fmicb.2020.00324 (PMC7066086; doi:10.3389/fmicb.2020.00324)
Supplement: Supplementary file 1 [file Presentation_1.PPTX]

## Slide 1
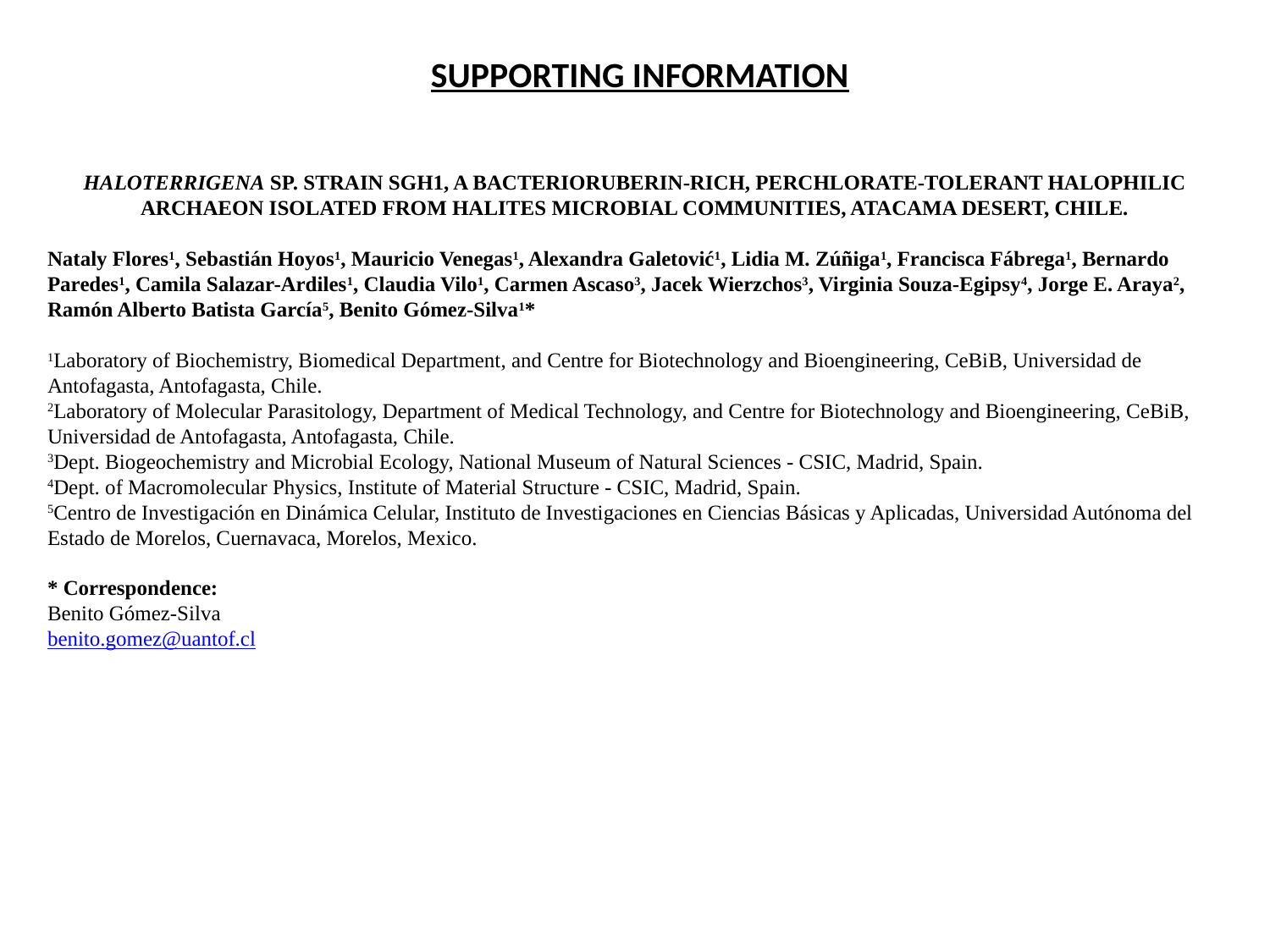

SUPPORTING INFORMATION
HALOTERRIGENA SP. STRAIN SGH1, A BACTERIORUBERIN-RICH, PERCHLORATE-TOLERANT HALOPHILIC ARCHAEON ISOLATED FROM HALITES MICROBIAL COMMUNITIES, ATACAMA DESERT, CHILE.
Nataly Flores1, Sebastián Hoyos1, Mauricio Venegas1, Alexandra Galetović1, Lidia M. Zúñiga1, Francisca Fábrega1, Bernardo Paredes1, Camila Salazar-Ardiles1, Claudia Vilo1, Carmen Ascaso3, Jacek Wierzchos3, Virginia Souza-Egipsy4, Jorge E. Araya2, Ramón Alberto Batista García5, Benito Gómez-Silva1*
1Laboratory of Biochemistry, Biomedical Department, and Centre for Biotechnology and Bioengineering, CeBiB, Universidad de Antofagasta, Antofagasta, Chile.
2Laboratory of Molecular Parasitology, Department of Medical Technology, and Centre for Biotechnology and Bioengineering, CeBiB, Universidad de Antofagasta, Antofagasta, Chile.
3Dept. Biogeochemistry and Microbial Ecology, National Museum of Natural Sciences - CSIC, Madrid, Spain.
4Dept. of Macromolecular Physics, Institute of Material Structure - CSIC, Madrid, Spain.
5Centro de Investigación en Dinámica Celular, Instituto de Investigaciones en Ciencias Básicas y Aplicadas, Universidad Autónoma del Estado de Morelos, Cuernavaca, Morelos, Mexico.
* Correspondence:
Benito Gómez-Silva
benito.gomez@uantof.cl

## Slide 2
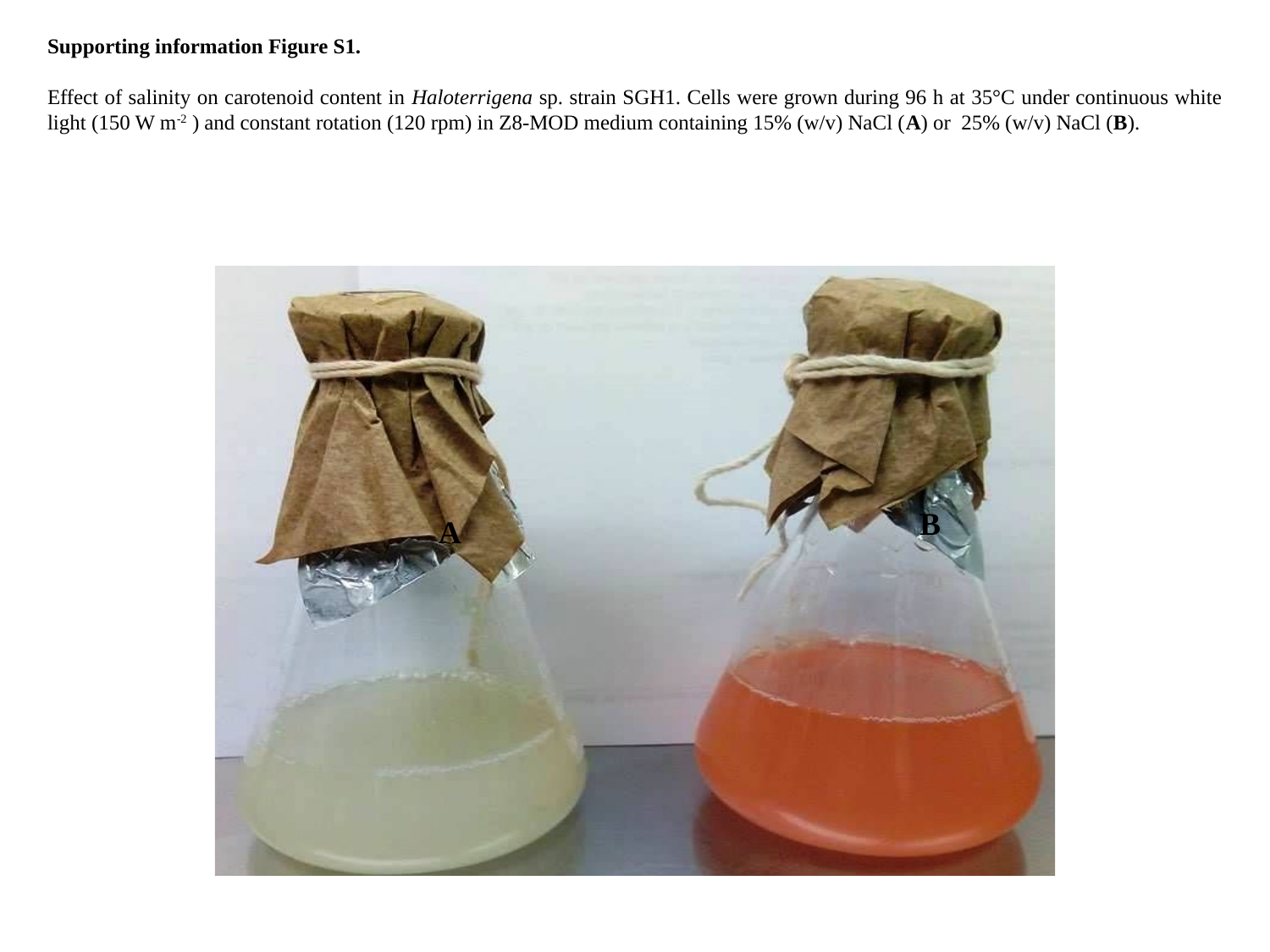

Supporting information Figure S1.
Effect of salinity on carotenoid content in Haloterrigena sp. strain SGH1. Cells were grown during 96 h at 35°C under continuous white light (150 W m-2 ) and constant rotation (120 rpm) in Z8-MOD medium containing 15% (w/v) NaCl (A) or 25% (w/v) NaCl (B).
B
A

## Slide 3
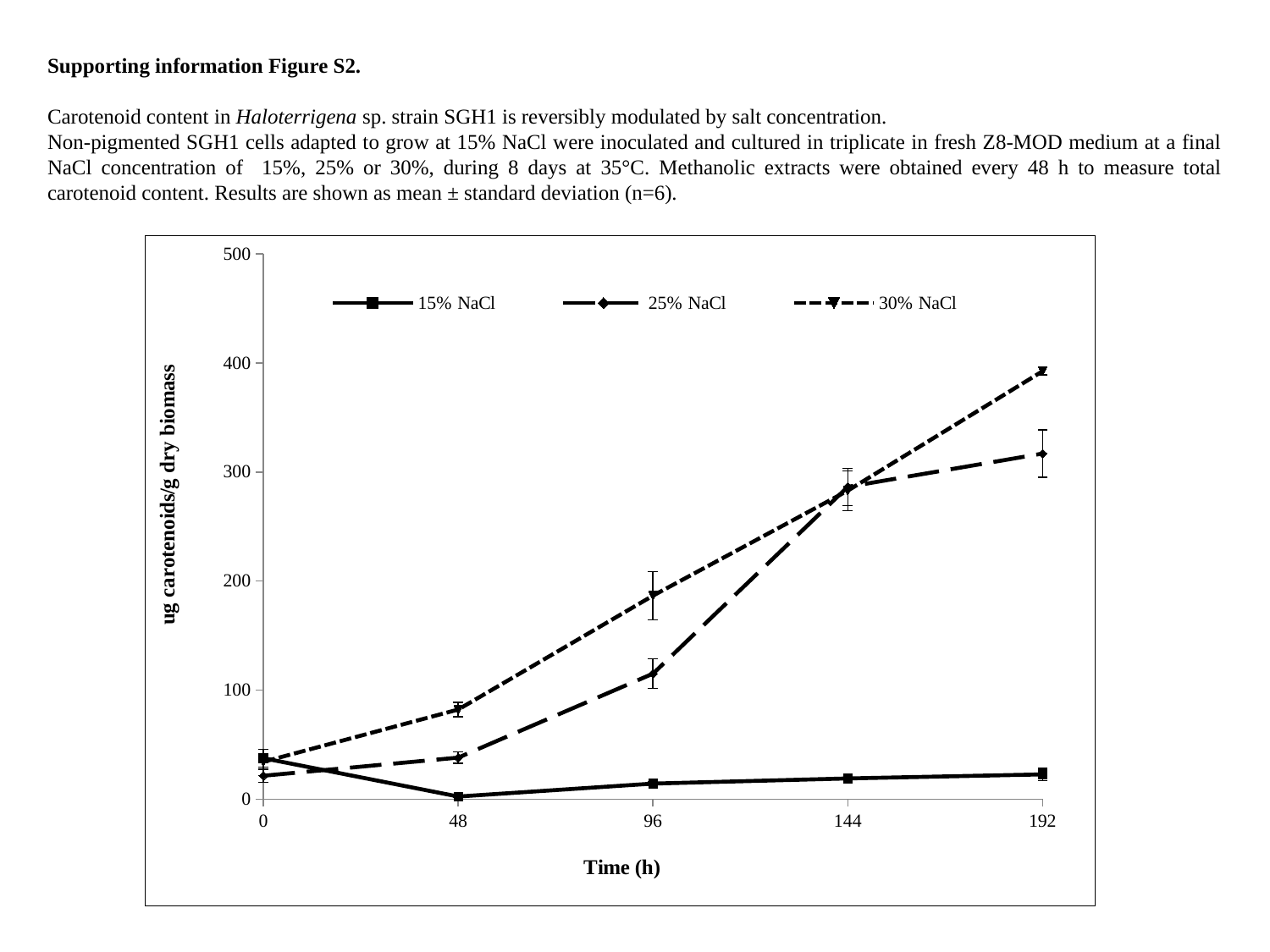

Supporting information Figure S2.
Carotenoid content in Haloterrigena sp. strain SGH1 is reversibly modulated by salt concentration.
Non-pigmented SGH1 cells adapted to grow at 15% NaCl were inoculated and cultured in triplicate in fresh Z8-MOD medium at a final NaCl concentration of 15%, 25% or 30%, during 8 days at 35°C. Methanolic extracts were obtained every 48 h to measure total carotenoid content. Results are shown as mean ± standard deviation (n=6).
### Chart
| Category | 15% NaCl | 25% NaCl | 30% NaCl |
|---|---|---|---|
| 0 | 37.578807676988106 | 21.51276269202664 | 34.293533350932954 |
| 48 | 2.44 | 38.13 | 82.23 |
| 96 | 14.326397518489356 | 115.14145882413538 | 186.66401475337193 |
| 144 | 19.023714466194924 | 286.45912378369866 | 282.99049550560085 |
| 192 | 22.768206997850807 | 317.0061156956177 | 392.54440421803525 |

## Slide 4
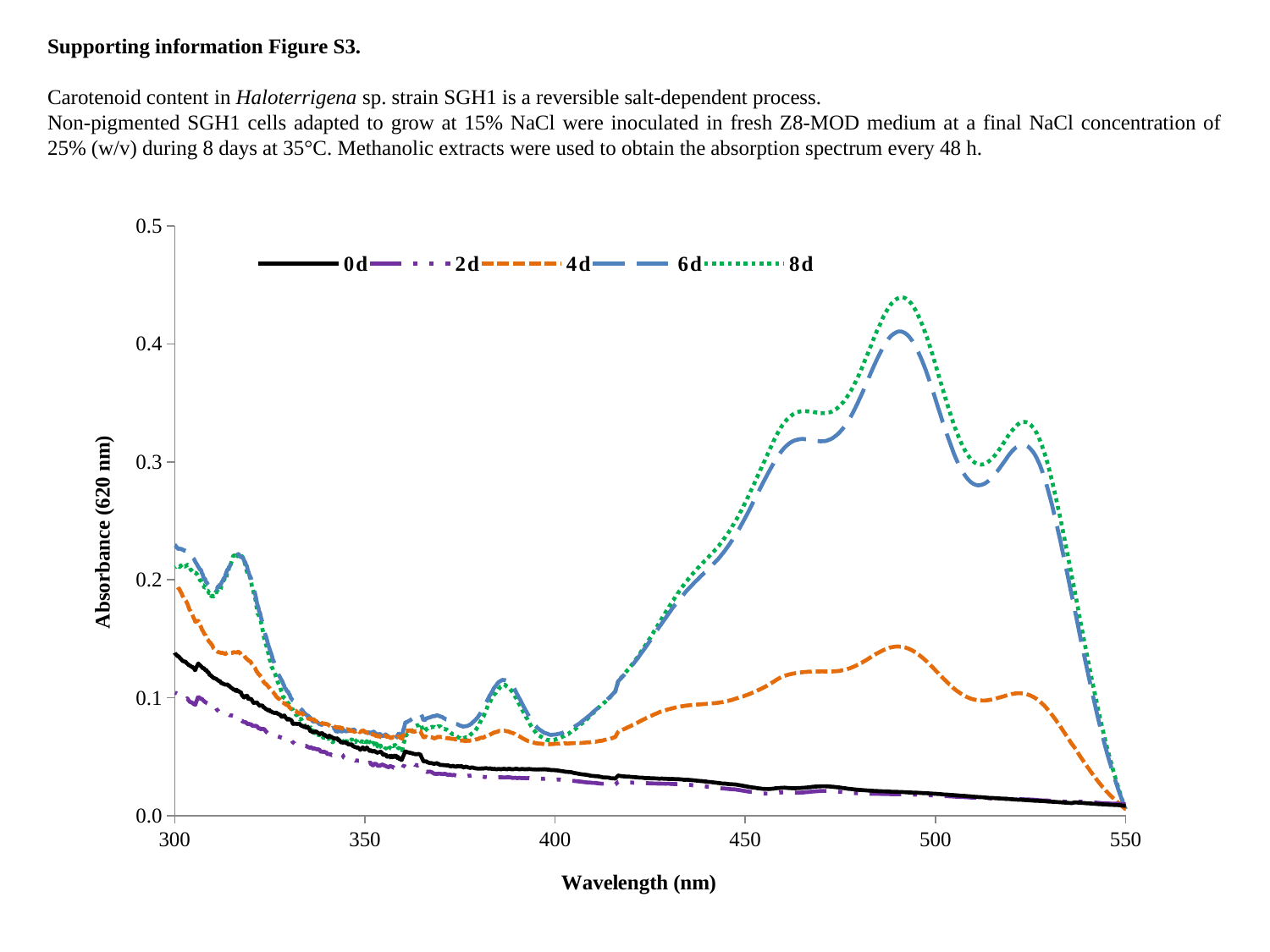

Supporting information Figure S3.
Carotenoid content in Haloterrigena sp. strain SGH1 is a reversible salt-dependent process.
Non-pigmented SGH1 cells adapted to grow at 15% NaCl were inoculated in fresh Z8-MOD medium at a final NaCl concentration of 25% (w/v) during 8 days at 35°C. Methanolic extracts were used to obtain the absorption spectrum every 48 h.
### Chart
| Category | 0d | 2d | 4d | 6d | 8d |
|---|---|---|---|---|---|

## Slide 5
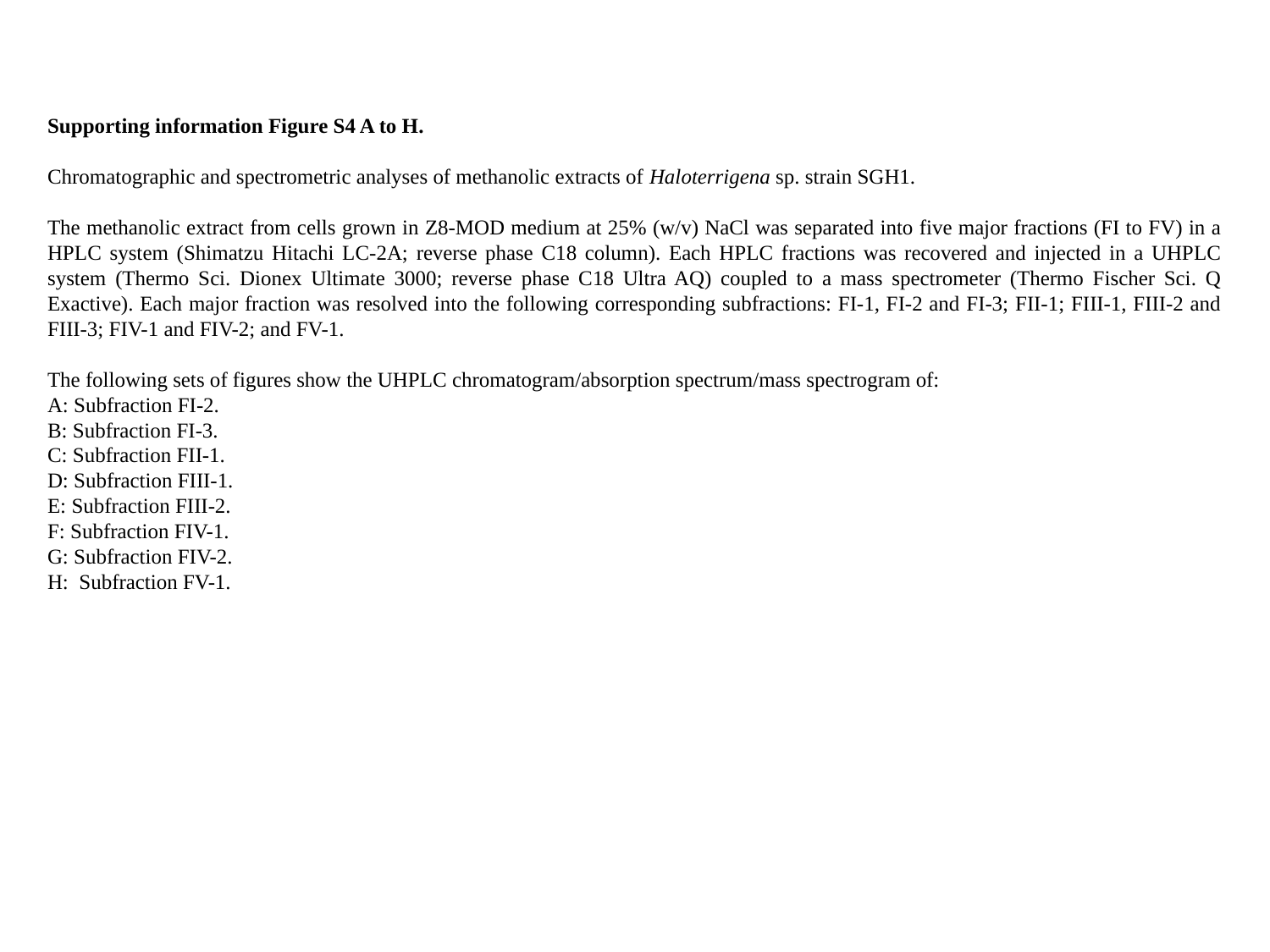

Supporting information Figure S4 A to H.
Chromatographic and spectrometric analyses of methanolic extracts of Haloterrigena sp. strain SGH1.
The methanolic extract from cells grown in Z8-MOD medium at 25% (w/v) NaCl was separated into five major fractions (FI to FV) in a HPLC system (Shimatzu Hitachi LC-2A; reverse phase C18 column). Each HPLC fractions was recovered and injected in a UHPLC system (Thermo Sci. Dionex Ultimate 3000; reverse phase C18 Ultra AQ) coupled to a mass spectrometer (Thermo Fischer Sci. Q Exactive). Each major fraction was resolved into the following corresponding subfractions: FI-1, FI-2 and FI-3; FII-1; FIII-1, FIII-2 and FIII-3; FIV-1 and FIV-2; and FV-1.
The following sets of figures show the UHPLC chromatogram/absorption spectrum/mass spectrogram of:
A: Subfraction FI-2.
B: Subfraction FI-3.
C: Subfraction FII-1.
D: Subfraction FIII-1.
E: Subfraction FIII-2.
F: Subfraction FIV-1.
G: Subfraction FIV-2.
H: Subfraction FV-1.

## Slide 6
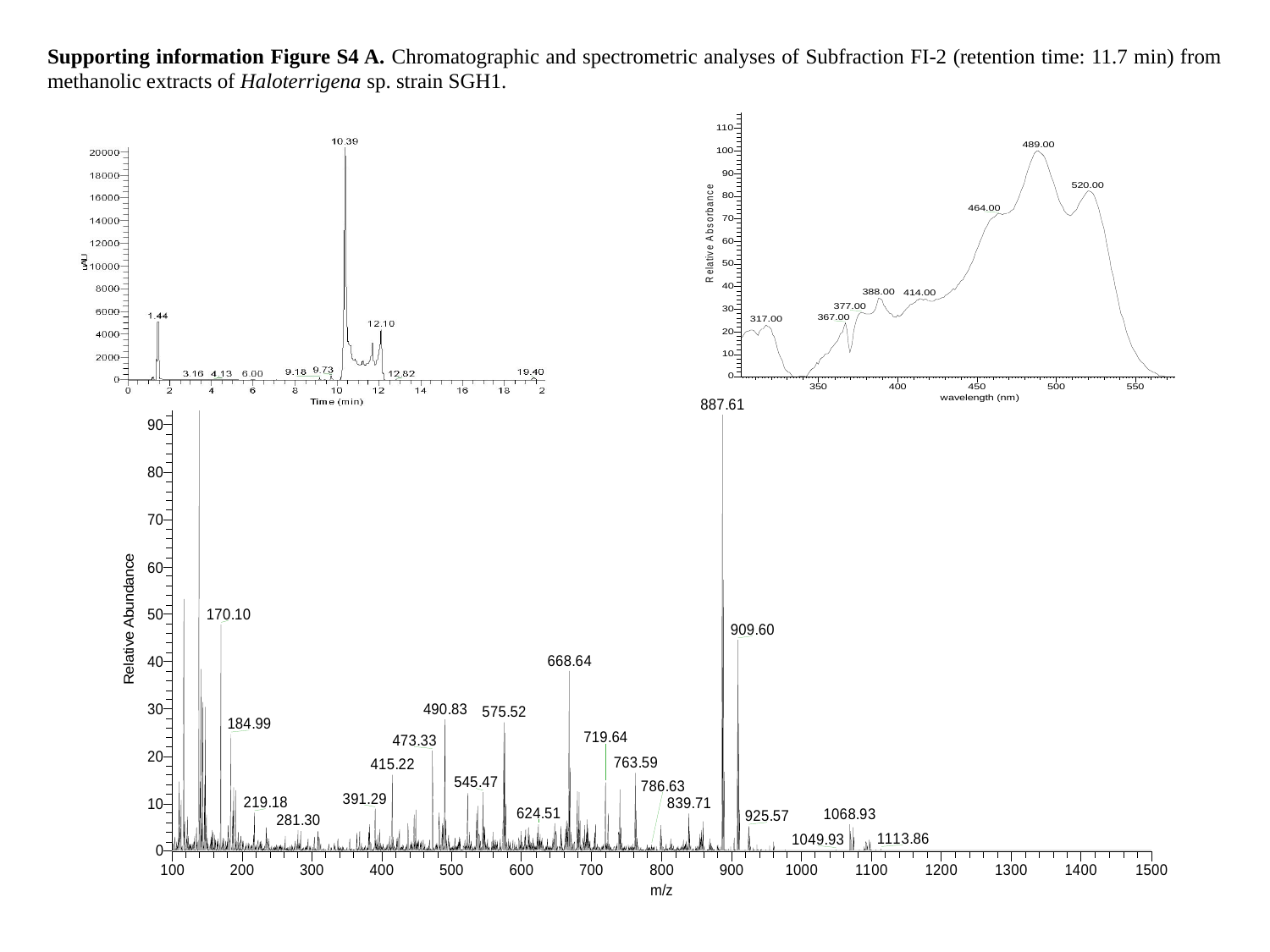

Supporting information Figure S4 A. Chromatographic and spectrometric analyses of Subfraction FI-2 (retention time: 11.7 min) from methanolic extracts of Haloterrigena sp. strain SGH1.

## Slide 7
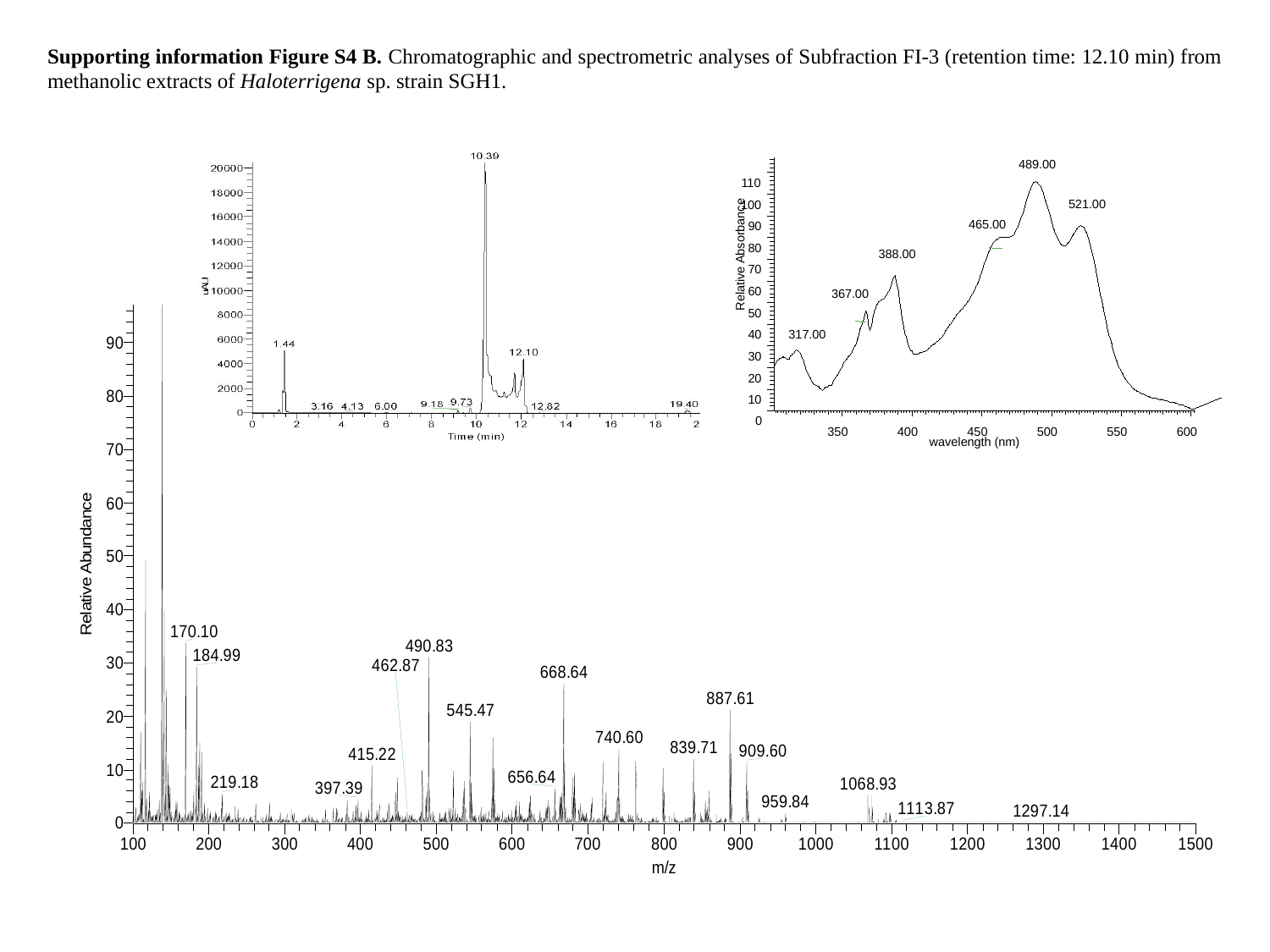

Supporting information Figure S4 B. Chromatographic and spectrometric analyses of Subfraction FI-3 (retention time: 12.10 min) from methanolic extracts of Haloterrigena sp. strain SGH1.
350
400
450
500
550
600
wavelength (nm)
489.00
110
521.00
100
465.00
90
80
388.00
Relative Absorbance
70
60
367.00
50
317.00
40
30
20
10
0

## Slide 8
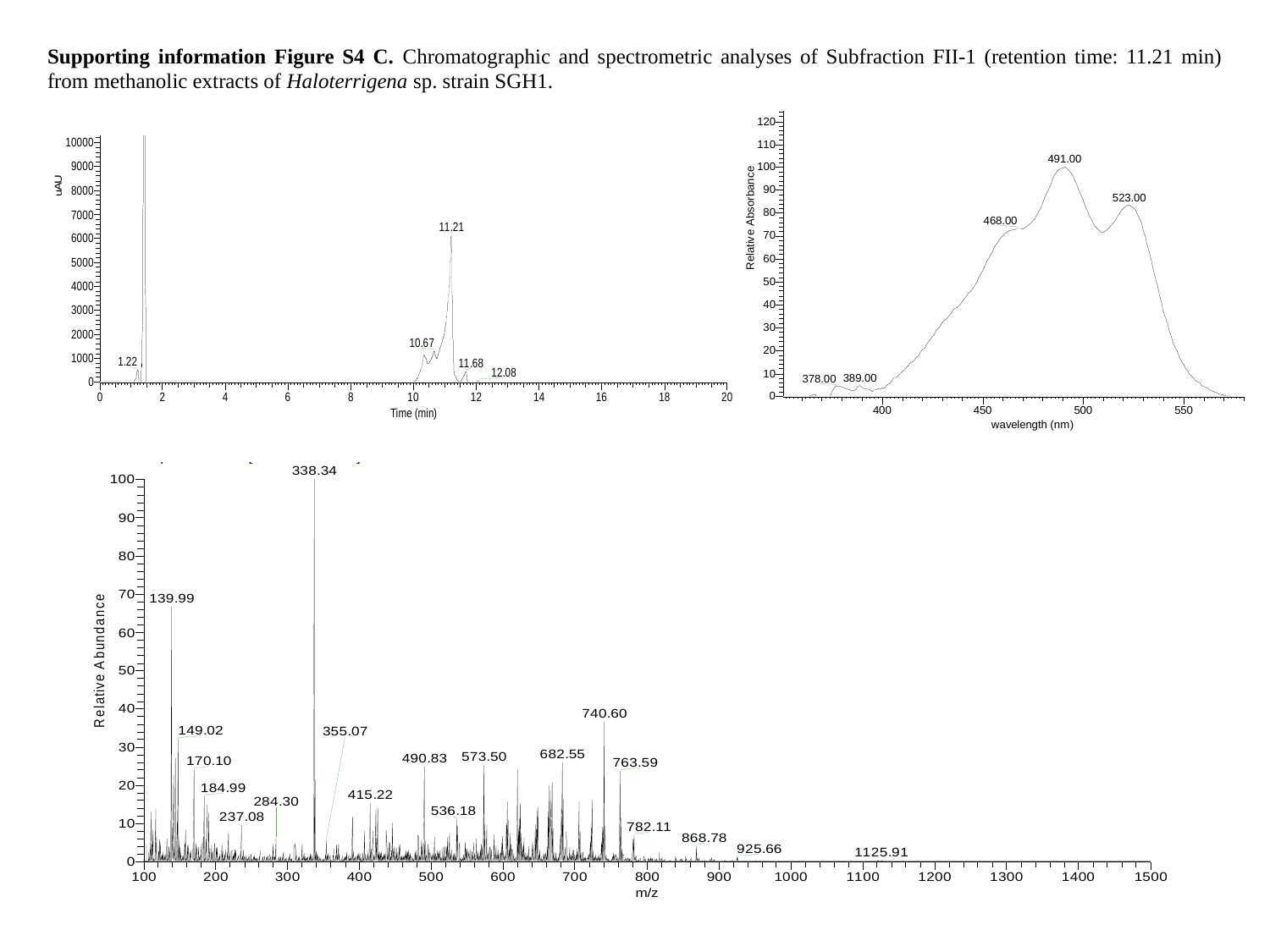

Supporting information Figure S4 C. Chromatographic and spectrometric analyses of Subfraction FII-1 (retention time: 11.21 min) from methanolic extracts of Haloterrigena sp. strain SGH1.

## Slide 9
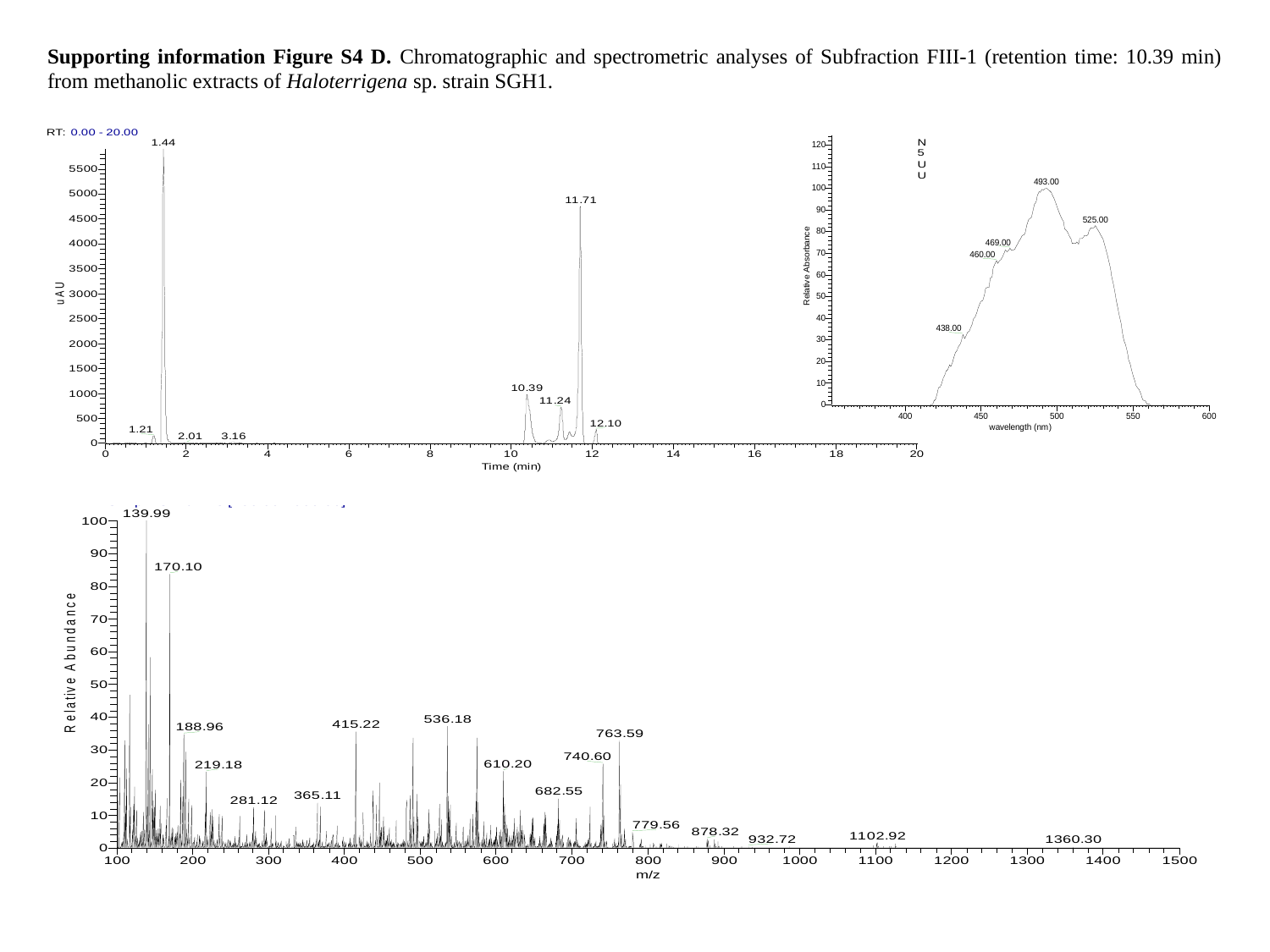

Supporting information Figure S4 D. Chromatographic and spectrometric analyses of Subfraction FIII-1 (retention time: 10.39 min) from methanolic extracts of Haloterrigena sp. strain SGH1.

## Slide 10
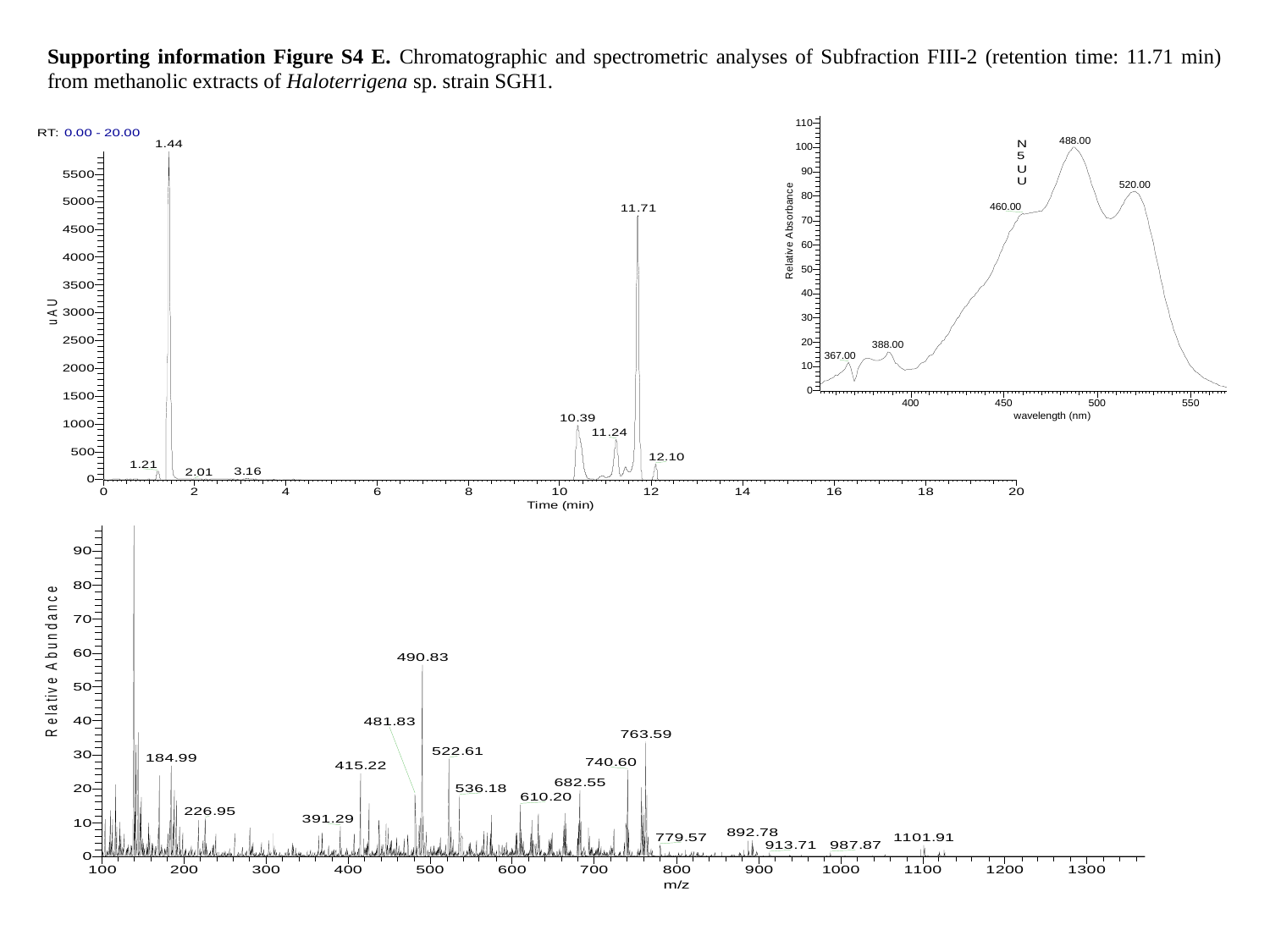

Supporting information Figure S4 E. Chromatographic and spectrometric analyses of Subfraction FIII-2 (retention time: 11.71 min) from methanolic extracts of Haloterrigena sp. strain SGH1.

## Slide 11
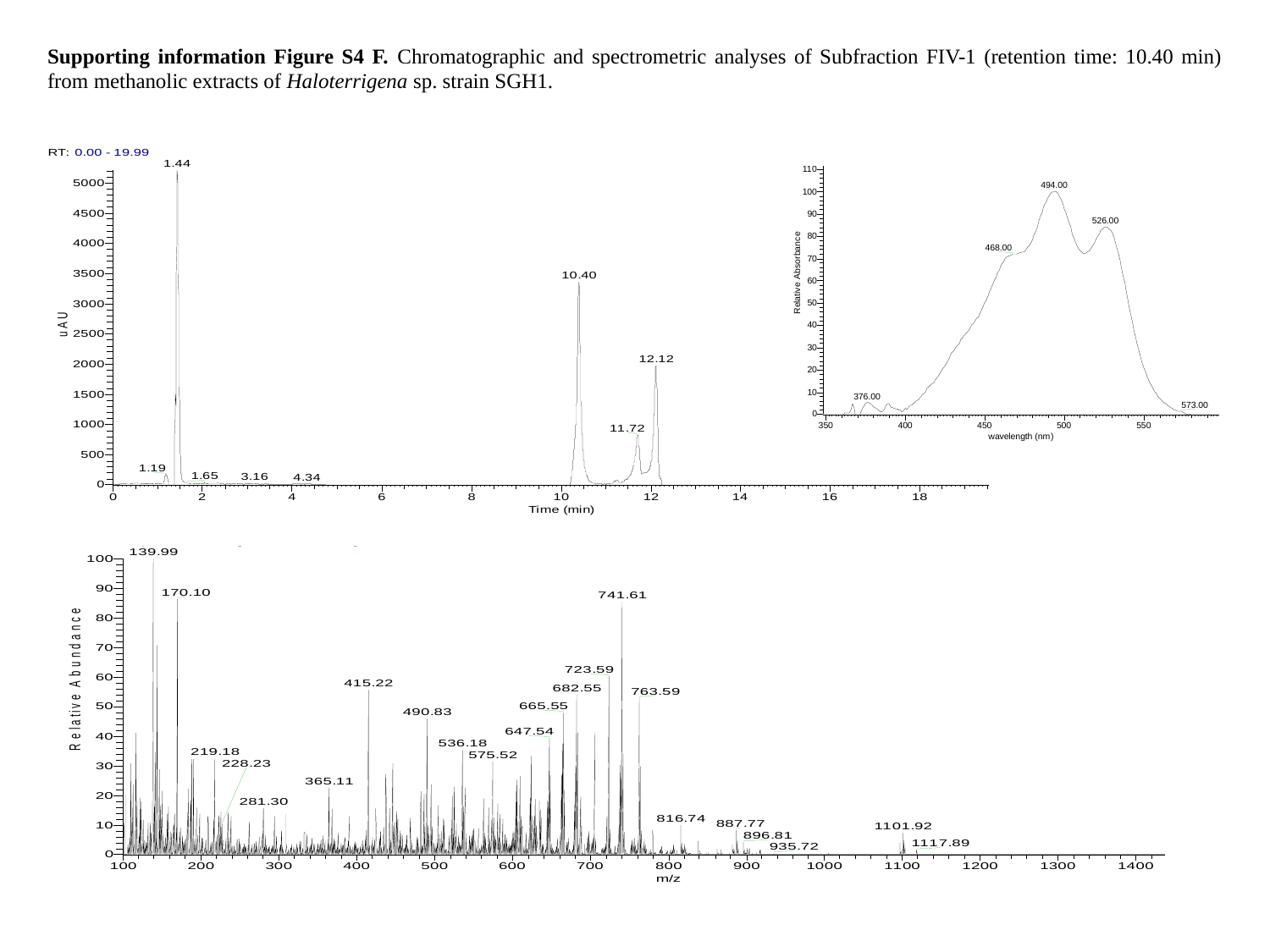

Supporting information Figure S4 F. Chromatographic and spectrometric analyses of Subfraction FIV-1 (retention time: 10.40 min) from methanolic extracts of Haloterrigena sp. strain SGH1.

## Slide 12
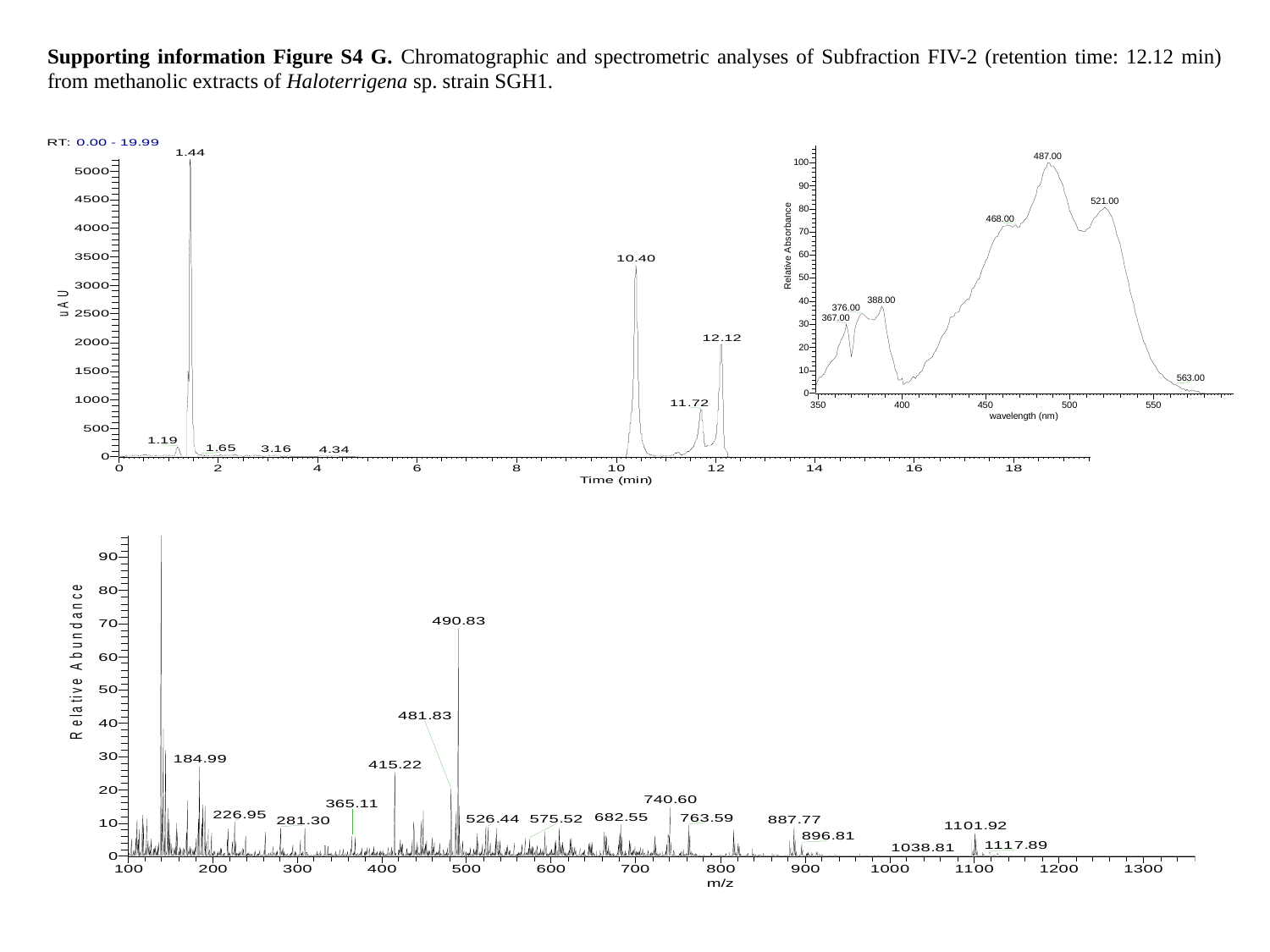

Supporting information Figure S4 G. Chromatographic and spectrometric analyses of Subfraction FIV-2 (retention time: 12.12 min) from methanolic extracts of Haloterrigena sp. strain SGH1.

## Slide 13
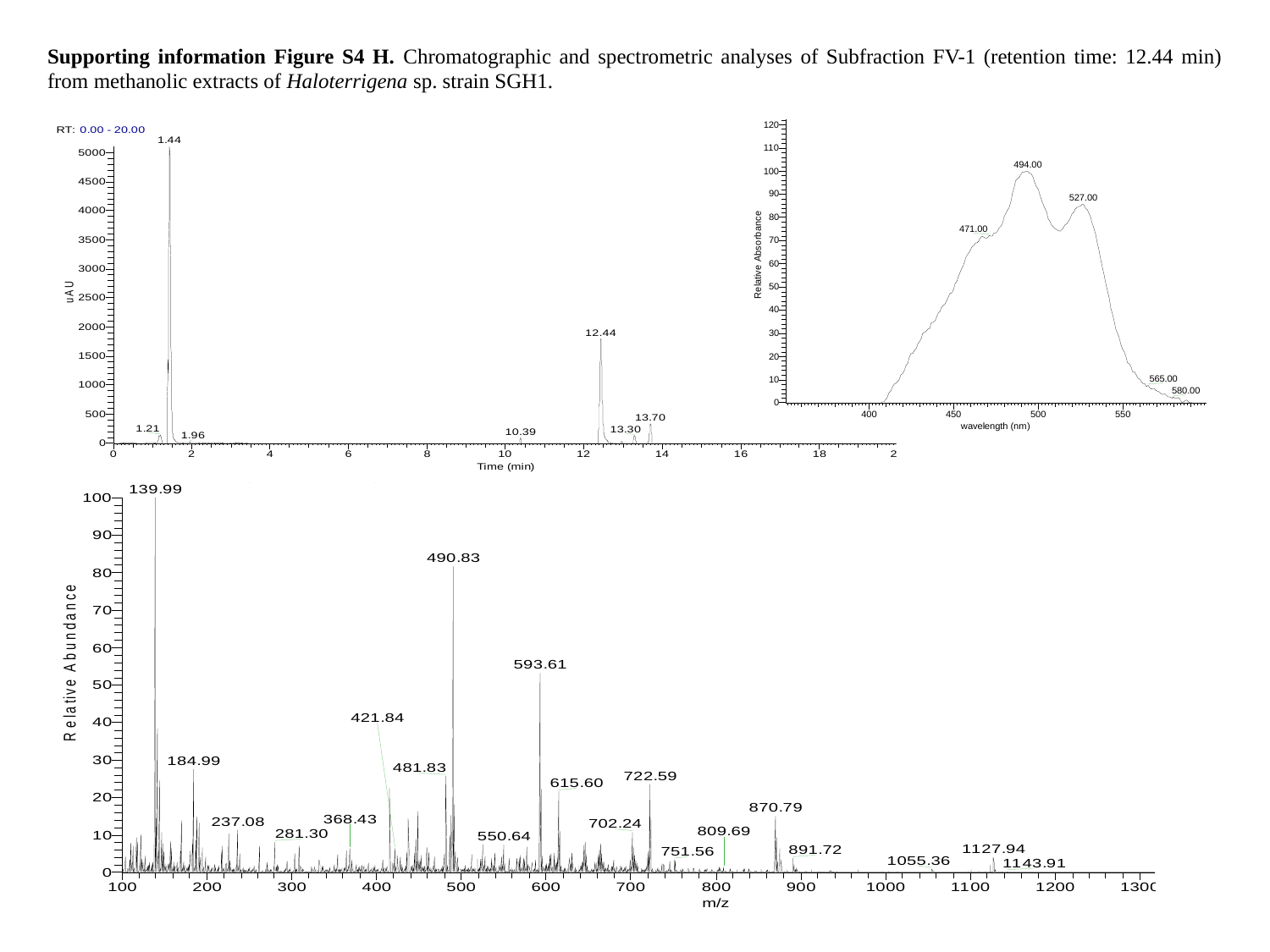

Supporting information Figure S4 H. Chromatographic and spectrometric analyses of Subfraction FV-1 (retention time: 12.44 min) from methanolic extracts of Haloterrigena sp. strain SGH1.

## Slide 14
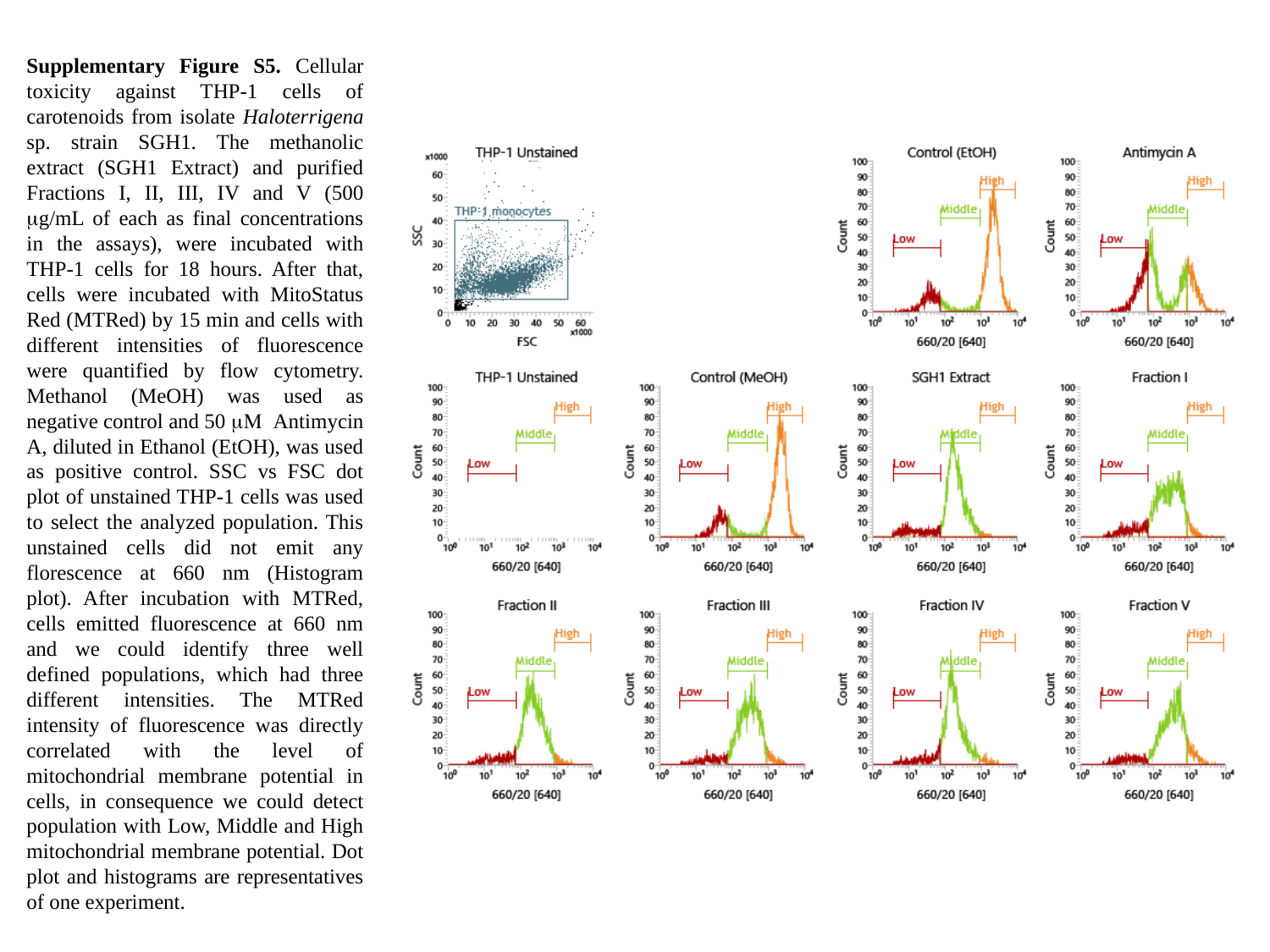

Supplementary Figure S5. Cellular toxicity against THP-1 cells of carotenoids from isolate Haloterrigena sp. strain SGH1. The methanolic extract (SGH1 Extract) and purified Fractions I, II, III, IV and V (500 μg/mL of each as final concentrations in the assays), were incubated with THP-1 cells for 18 hours. After that, cells were incubated with MitoStatus Red (MTRed) by 15 min and cells with different intensities of fluorescence were quantified by flow cytometry. Methanol (MeOH) was used as negative control and 50 μM Antimycin A, diluted in Ethanol (EtOH), was used as positive control. SSC vs FSC dot plot of unstained THP-1 cells was used to select the analyzed population. This unstained cells did not emit any florescence at 660 nm (Histogram plot). After incubation with MTRed, cells emitted fluorescence at 660 nm and we could identify three well defined populations, which had three different intensities. The MTRed intensity of fluorescence was directly correlated with the level of mitochondrial membrane potential in cells, in consequence we could detect population with Low, Middle and High mitochondrial membrane potential. Dot plot and histograms are representatives of one experiment.
