# Supplementary material for: Haloterrigena sp. Strain SGH1, a Bacterioruberin-Rich, Perchlorate-Tolerant Halophilic Archaeon Isolated From Halite Microbial Communities, Atacama Desert, Chile
Source: Front Microbiol. 2020 Mar 5;11:324. doi: 10.3389/fmicb.2020.00324 (PMC7066086; doi:10.3389/fmicb.2020.00324)
Supplement: Supplementary file 2 [file Table_1.docx]

**SUPPORTING INFORMATION**

***HALOTERRIGENA* SP. STRAIN SGH1, A BACTERIORUBERIN-RICH, PERCHLORATE-TOLERANT HALOPHILIC ARCHAEON ISOLATED FROM HALITES MICROBIAL COMMUNITIES, ATACAMA DESERT, CHILE.**

Nataly Flores^1^, Sebastián Hoyos^1^, Mauricio Venegas^1^, Alexandra Galetović^1^, Lidia M. Zúñiga^1^, Francisca Fábrega^1^, Bernardo Paredes^1^, Camila Salazar-Ardiles^1^, Claudia Vilo^1^, Carmen Ascaso^3^, Jacek Wierzchos^3^, Virginia Souza-Egipsy^4^, Jorge E. Araya^2^, Benito Gómez-Silva^1^*

^1^Laboratory of Biochemistry, Biomedical Department, and Centre for Biotechnology and Bioengineering, CeBiB, Universidad de Antofagasta, Antofagasta, Chile.

^2^Laboratory of Parasitology and Molecular Biology, Department of Parasitology, and Centre for Biotechnology and Bioengineering, CeBiB, Universidad de Antofagasta, Antofagasta, Chile.

^3^Dept. Biogeochemistry and Microbial Ecology, National Museum of Natural Sciences - CSIC, Madrid, Spain.

^4^Dept. of Macromolecular Physics, Institute of Material Structure - CSIC, Madrid, Spain.

*** Correspondence:**Benito Gómez-Silva
[benito.gomez@uantof.cl](mailto:benito.gomez@uantof.cl)

**Table S1**. Database for the identification and relative abundance of bacterioruberin molecules from *Haloterrigena* sp. strain SGH1.

| **Fraction** | **Sub-fractions** | **RT (min)** | **λ max (nm)** | | | | **A^cis^/A^II^***  **(%)** | **A^III^/A^II^***  **(%)** | **Molecular Ion (M+H)^+^ (m/z)** | **MS fragments**  **(m/z)** | **Carotenoid**** | **Relative abundance (%)** |
| --- | --- | --- | --- | --- | --- | --- | --- | --- | --- | --- | --- | --- |
|  |  |  | **Cis** | **I** | **II** | **III** |  |  |  |  |  |  |
| I | F-I.1 | 10.4 | 388 | 457 | 493 | 526 | 22 | 81 | 741.6 | 724, 706, 688, 684, 666, 648, 630, 582, 576, 425 | all-*trans*-BR | 43.3 |
|  | F-I.2 | 11.7 | 388 | 464 | 489 | 520 | 35 | 77 | 668.6 | 666, 577, 546, 538, 492, 447 | all-*trans*-TABR | 3.4 |
|  | F-I.3 | 12.1 | 388 | 464 | 489 | 521 | 58 | 82 | 668.6 | 665, 577, 546, 538, 492, 447, 369 | *cis*-TABR | 8.3 |
| II | F-II.1 | 11.2 | 388 | 468 | 491 | 523 | 9 | 83 | 740.6 | 723, 706, 683, 669, 665, 648, 578, 538, 447, 426 | 5-*cis*-BR | 15 |
| III | F-III.1 | 10.4 |  | 466 | 493 | 527 | 0 | 81 | 740.6 | 724, 683, 665, 610, 576, 536, 512, 497, 453, 447, 443, 425, 369 | all-*trans*-BR | 2.6 |
|  | F-III.2 | 11.7 | 388 | 460 | 488 | 520 | 16 | 85 | 740.6 | 683, 665, 610, 576, 536, 492, 489, 447, 426, 369 | 9-*cis*-BR | 10.4 |
| IV | F-IV.1 | 10.4 | 388 | 468 | 494 | 526 | 6 | 85 | 741.6 | 724, 706, 683, 666, 648, 576, 536, 453, 447, 369 | all-*trans*-BR | 3.7 |
|  | F-IV.2 | 12.1 | 388 | 463 | 487 | 521 | 40 | 80 | 740.6 | 723, 610, 683, 369, 453, 492, 536, 664, 489, 447 | 13-*cis*-BR | 4.3 |
| V | F-V.1 | 12.4 |  | 467 | 494 | 527 | 0 | 93 | 722.6 | 594, 723, 492, 664, 647, 489, 369, 617, 578, 447 | all-*trans*-MABR | 9.0 |

*A^cis^: 388 nm; A^II^: 487-494 nm; A^III^: 521-527 nm; **BR: bacterioruberin; MABR: mono-anhydrobacterioruberin; BABR: bis-anhydrobacterioruberin; TABR: tetra-anhydrobacterioruberin.
